# EHD2 regulates plasma membrane integrity and downstream insulin receptor signaling events

**DOI:** 10.1091/mbc.E23-03-0078

**Published:** 2023-10-31

**Authors:** Mathis Neuhaus, Claes Fryklund, Holly Taylor, Andrea Borreguero-Muñoz, Franziska Kopietz, Hamidreza Ardalani, Oksana Rogova, Laura Stirrat, Shaun K. Bremner, Peter Spégel, Nia J. Bryant, Gwyn W. Gould, Karin G. Stenkula

**Affiliations:** a Department of Experimental Medical Science, Lund University, 22184 Lund, Sweden; b Strathclyde Institute for Pharmacy and Biomedical Sciences, University of Strathclyde, Glasgow G4 0RE, UK; c Department of Chemistry, Centre for Analysis and Synthesis, Lund University, 22241 Lund, Sweden; d Department of Biology and York Biomedical Research Institute, University of York, York YO10 5DD, UK; University of Pittsburgh Medical School

## Abstract

Adipocyte dysfunction is a crucial driver of insulin resistance and type 2 diabetes. We identified EH domain-containing protein 2 (EHD2) as one of the most highly upregulated genes at the early stage of adipose-tissue expansion. EHD2 is a dynamin-related ATPase influencing several cellular processes, including membrane recycling, caveolae dynamics, and lipid metabolism. Here, we investigated the role of EHD2 in adipocyte insulin signaling and glucose transport. Using C57BL6/N EHD2 knockout mice under short-term high-fat diet conditions and 3T3-L1 adipocytes we demonstrate that EHD2 deficiency is associated with deterioration of insulin signal transduction and impaired insulin-stimulated GLUT4 translocation. Furthermore, we show that lack of EHD2 is linked with altered plasma membrane lipid and protein composition, reduced insulin receptor expression, and diminished insulin-dependent SNARE protein complex formation. In conclusion, these data highlight the importance of EHD2 for the integrity of the plasma membrane milieu, insulin receptor stability, and downstream insulin receptor signaling events, involved in glucose uptake and ultimately underscore its role in insulin resistance and obesity.

## Abstract (executive summary)

EHD2 is a dynamin-related ATPase involved in several cellular processes, including membrane recycling, caveolae dynamics, and lipid metabolism.

Our results demonstrate that EHD2 deficiency modulates membrane lipid composition and negatively influences insulin receptor stability and downstream events, including SNARE protein complex formation, GLUT4 dynamics, and glucose uptake using primary and cultured adipocyte models.

These findings pinpoint that EHD2 has an essential role in sustaining plasma membrane integrity and events involved in insulin signal transduction and glucose uptake.

## INTRODUCTION

Obesity is one of the most serious challenges for global health in the 21st century. In the United States, the obesity prevalence rate has increased from 30.5 to 42.4% the last 20 y, and excess weight is a well-established risk factor for numerous diseases, including insulin resistance and type 2 diabetes. Overweight and obesity are characterized by impaired adipocyte function, involving reduced insulin sensitivity, lowered glucose uptake, and impaired lipid storage capacity, which negatively influences whole-body glucose homeostasis (Acosta *et al.*, 2016).

Caveolae are omega-shaped plasma membrane (PM) invaginations with a broad range of functions, for example, the orchestration of lipid transport (Pohl *et al.*, 2005), facilitation of transmembrane signaling (Cohen *et al.*, 2003), and stabilization of the insulin receptor (IR; Gustavsson *et al.*, 1999). Approximately one-third of the adipocyte cell surface area is occupied by caveolae (Thorn *et al.*, 2003), and mounting evidence implies that caveolae capture a critical function in maintaining proper insulin signaling in adipocytes (reviewed in Stralfors, 2012). Ablation or mutation of the caveolae core proteins, namely cavins and caveolins, leads to impaired insulin signaling in adipose tissue, hyperinsulinemia, and insulin resistance (Gustavsson *et al.*, 1999; Cohen *et al.*, 2003), and lipodystrophy in both rodent models (Razani *et al.*, 2002; Liu *et al.*, 2008) and humans (Cao *et al.*, 2008; Kim *et al.*, 2008). Further, Cohen *et al.* (2003) reported an increased proteasomal degradation of insulin receptor beta (IRβ) and insulin receptor substrate (IRS-1) in caveolin-1 (CAV1)-deficient mice. Consequently, caveolae structures potentially stabilize the IR, govern insulin signal transduction, and exert significant effects on insulin-dependent processes in the adipocytes. Consistent with this, caveolae-mediated endocytosis of the IR drives dephosphorylation of the autophosphorylated IR and thus ensures proper insulin signaling (Fagerholm *et al.*, 2009; Chen *et al.*, 2019). However, the exact interplay of caveolae and insulin signaling remains to be elucidated. One possible way caveolae might influence insulin signaling could be related to the unique lipid environment of caveolae structures that are enriched in cholesterol, sphingomyelin (SM), and phosphatidylethanolamine (PE); indeed, previous studies indicate that both the activity and stability of IRβ are affected by membrane lipid alterations (Ginsberg *et al.*, 1981; Vainio *et al.*, 2002).

EH domain-containing protein 2 (EHD2) is a dynamin-related ATPase (Daumke *et al.*, 2007) that oligomerizes at the neck of caveolae where it is proposed to stabilize caveolae at the PM through cytoskeletal anchoring (Moren *et al.*, 2012; Stoeber *et al.*, 2012). This was illustrated by EHD2 knockdown (KD) which caused increased caveolae detachment (Moren *et al.*, 2012; Matthaeus *et al.*, 2020). Following a short-term high-fat diet (HFD) feeding intervention, we discovered *EHD2* as one of the most differentially expressed genes in adipose tissue (Hansson *et al.*, 2018). In recent studies, we and others have confirmed a role for EHD2 in lipid handling using a global EHD2 knockout (EHD2 KO) C57Bl6/N mouse model, which displayed altered lipid metabolism (Matthaeus *et al.*, 2020; Fryklund *et al.*, 2021), impaired lipolysis, and diminished insulin-mediated inhibition of lipolysis in primary adipocytes (Fryklund *et al.*, 2021). Interestingly, it was previously shown that antibody-induced EHD2 inhibition reduced insulin-stimulated GLUT4 translocation (Park *et al.*, 2004), emphasizing a role of EHD2 for insulin-dependent cellular events. A key mechanism regulating the insulin-stimulated delivery of intracellular GLUT4 sortage vesicles (GSV) to the PM is the assembly of soluble N-ethylmaleimide-sensitive factor-attachment protein receptor (SNARE) protein complexes (Bryant *et al.*, 2002). The structure of individual SNARE proteins (Saito *et al.*, 2012; Fezoua-Boubegtiten *et al.*, 2019; Lakomek *et al.*, 2019; Wang *et al.*, 2020), their localization (Chamberlain *et al.*, 2001; Lang *et al.*, 2001; Salaun *et al.*, 2005), and their assembly has been shown to depend on the membrane lipid environment (Schnitzer *et al.*, 1995; Chamberlain and Gould, 2002). Accordingly, due to the exceptionally high abundance in adipocytes and the unique lipid composition of caveolae, it could be postulated that SNARE function might be affected by caveolae integrity. However, to the best of our knowledge, the impact of EHD2 deficiency on insulin signaling and SNARE protein complex formation has not been elucidated.

Therefore, we set out to examine the effects of EHD2 deficiency on insulin signaling, glucose uptake, and PM lipid composition using primary adipocytes isolated from C57BL6/N EHD2 KO mice. In addition, by siRNA-induced EHD2 KD in cultured 3T3-L1 adipocytes, we examined the role of EHD2 in SNARE complex assembly and CAV1–IRβ interactions. We demonstrate that EHD2 deficiency negatively influences IRβ stability and downstream events, including SNARE protein complex formation, GLUT4 dynamics, and glucose uptake and posit that EHD2 plays a key role in the organization and integrity of insulin signaling and PM composition.

## RESULTS

### EHD2 deficiency impairs GLUT4 translocation and glucose uptake in adipocytes

**FIGURE 1:**
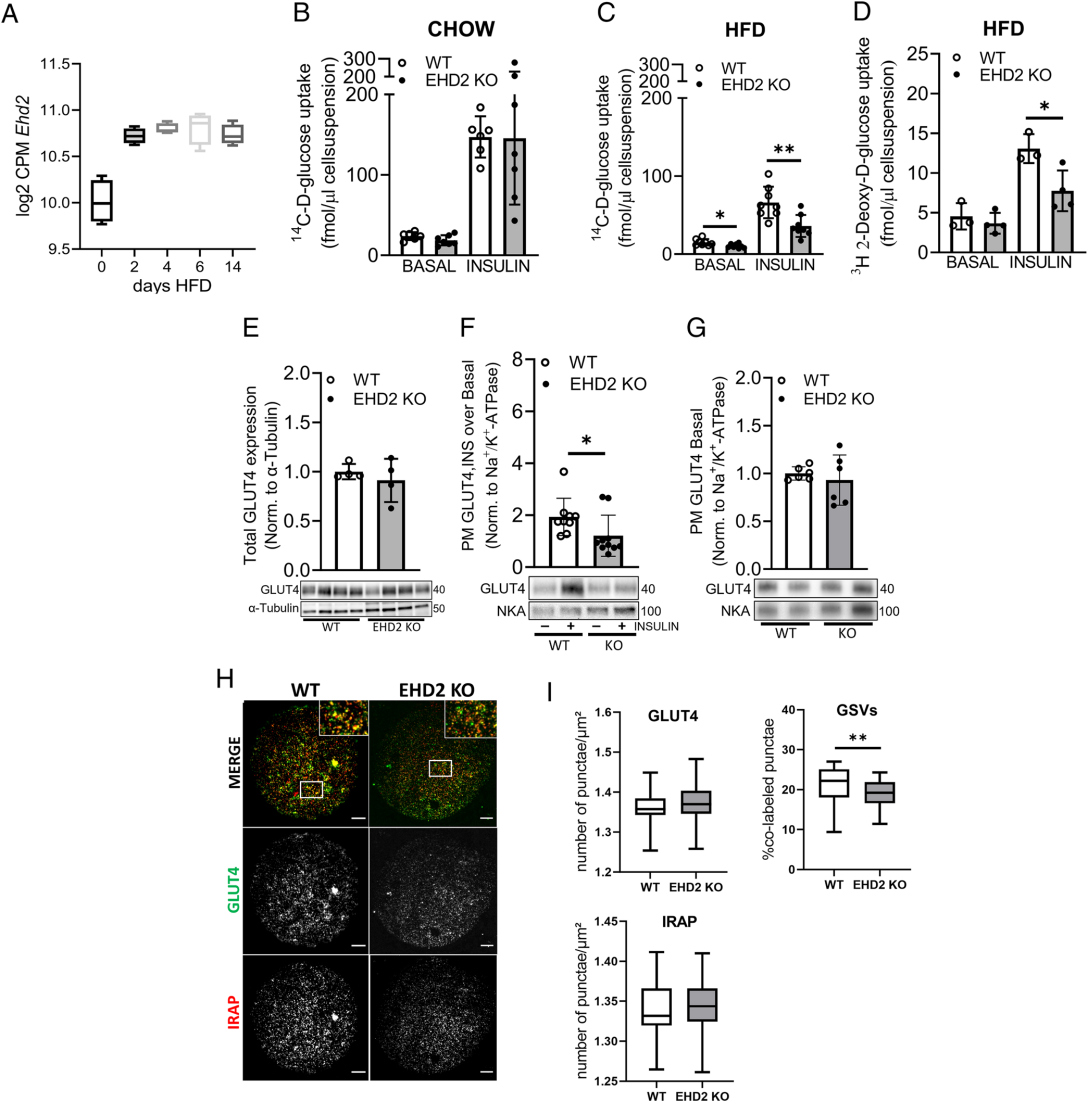
Insulin-stimulated glucose uptake and GLUT4 dynamics of EHD2-deficient adipocytes. (A) RNA expression of *Ehd2* at d 0 (chow), 2, 4, 6, and 14 d of HFD in epididymal adipose tissue. Average expression of *n* = 4 per group is displayed in log “counts per million”. B–I refers to data collected from primary inguinal adipocytes isolated from WT and EHD2 KO mice. Non- (basal) and insulin-stimulated (1 nM, 30 min) glucose uptake in primary inguinal adipocytes isolated from chow-fed (B) or HFD-fed (C) mice using ^14^C-glucose tracer assay, *n* = 6-8 biological replicates. (D) [3H]2-Deoxy-d-glucose (2-DG) uptake in non- (basal) and insulin-stimulated (1 nM, 30 min) from HFD-fed mice, *n* = 3-4 biological replicates. (E) GLUT4 protein levels in nonstimulated whole-cell lysates; each lane represents a biological replicate, *n* = 4. (F) Insulin-stimulated (1 nM, 30 min) GLUT4 translocation in HFD inguinal adipocytes using PM sedimentation assay (Nishiumi and Ashida, 2007). Data are displayed as fold change of insulin over basal. *n* = 9 and 10, statistical comparison was carried out using Mann-Whitney test (*p ≤ 0.05). (G) GLUT4 protein levels in nonstimulated (basal) PM fractions, *n* = 5 biological replicates. (H) Representative TIRF images of inguinal adipocytes isolated from WT and EHD2 KO mice; (35 cells/replicate). Scale bar = 10 µm. Cells were colabeled with GLUT4 (green) and IRAP (red) antibodies. (I) Number of GLUT4-positive punctae/µm^2^, number of IRAP-positive punctae/µm^2^ and quantification of colabeled punctae (GLUT4 and IRAP) below 140 nm, expressed as percentage of all detected GLUT4 puncta (defined as GSVs). If not stated otherwise, unpaired two-sample *t* test was used for statistical analysis. Data are displayed as mean ± SD and significance was determined according to *p ≤ 0.05 and **p ≤ 0.01. All displayed results, except for A and B, were obtained from inguinal adipocytes after 2 wk of HFD. NKA = Na^+^/K^+^-ATPase.

Previously, we identified EHD2 as one of the highest upregulated genes in white adipose tissue in response to short-term HFD feeding (Figure 1A; data retrieved from RNA sequencing performed in a previous study [Hansson *et al.*, 2018]). This finding suggests that EHD2 plays a key role during the development of overweight and obesity. Accordingly, we chose to monitor the impact of EHD2 deficiency on cellular glucose transport, both in the chow-fed state as well as after 2 wk of HFD to resolve possible differences related to the degree of adipose tissue expansion. Therefore, we analyzed glucose uptake in primary inguinal adipocytes isolated from EHD2 KO mice and corresponding control (WT) mice. While both non- and insulin-stimulated glucose uptake were similar in EHD2 KO and WT adipocytes in the chow-fed state (Figure 1B), we found a significant reduction in both basal- and insulin-stimulated glucose uptake (∼30% basal and ∼45% insulin) in EHD2 KO adipocytes compared with WT adipocytes isolated from HFD-fed mice (Figure 1C). This finding was confirmed using a nonmetabolizable 2-Deoxy-D-glucose tracer, the data of which indicates that glucose uptake, rather than glucose metabolism, is reduced in EHD2-deficient adipocytes (Figure 1D). Taken together, lack of EHD2 affected glucose uptake in the obese but not chow-fed state. As EHD2 expression is highly upregulated during HFD and differences in glucose uptake are only observable in adipocytes from HFD-fed mice, we focused on examining the mechanisms of EHD2 in adipocytes isolated after short-term HFD feeding.

Total cellular GLUT4 levels were similar in whole-cell lysates of WT and EHD2 KO adipocytes in the HFD-fed animals (Figure 1E). However, and consistent with glucose transport assays, the ability of insulin to increase PM GLUT4 levels was markedly lower in EHD2 KO adipocytes (Figure 1F). These data argue for an impairment in insulin-stimulated GLUT4 translocation in EHD2 KO adipocytes despite normal levels of total cellular GLUT4 in these animals. Intriguingly, we observed similar fold increase of IRAP in the PM of both WT and EHD2 KO adipocytes after insulin stimulation (Supplemental Figure S1A). Further, TIRF microscopy demonstrated that EHD2 KO adipocytes display lowered GSV number in the basal state (Figure 1, H and I). Hence, the lower basal rate of glucose transport in EHD2 KO cells could be related to changes in GSV formation as well as expression of other GLUT isoforms such as GLUT1, which is known to contribute to basal glucose uptake. This is supported by the notion that we observed similar levels of GLUT4 in the basal state in WT and EHD2 KO cells using PM sedimentation assay (Figure 1G) and TIRF imaging (Figure 1I). Furthermore, we observed that basal IRAP levels in PM proximity are similar in WT and EHD2 KO accessed by both PM sedimentation assay (Supplemental Figure 1, A [right]) and TIRF imaging (Figure 1I).

### Reduced caveolae protein expression, IR stability, and downstream insulin signaling in EHD2 KO cells

**FIGURE 2:**
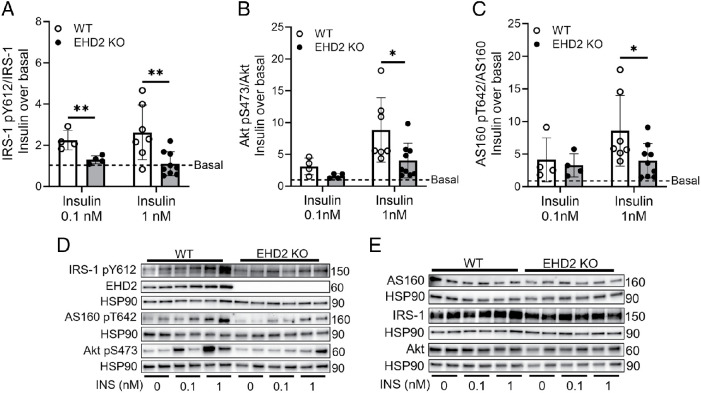
Impaired insulin signaling in EHD2 KO adipocytes. Inguinal adipocytes were nonstimulated or stimulated with insulin (0.1 or 1 nM) for 30 min, followed by Western blot analysis to detect total and phosphorylated protein levels of (A) IRS-1 (pY612), (B) AKT (pS473), (C) AS160 (pT642) and EHD2. Data are presented as fold change of insulin over basal (basal = 1, displayed as dashed line). Representative Western blots of phosphorylated protein levels (D) and total protein levels (E) of two biological replicates. Basal (phosphorylated/total) protein levels are available in Supplemental Figure S1B. Data are displayed as mean ± SD. Statistical comparison was carried out using using Mann-Whitney test (1 nM Akt S473 and 1 nM AS160 Thr642) or unpaired two-sample *t* test. Significance was determined according to *p ≤ 0.05 and **p ≤ 0.01. Representative blots from *n* = 4–9 biological replicates. All displayed results were obtained from inguinal adipocytes 2 wk of HFD.

Next, we examined whether impaired insulin signal transduction could explain the lowered glucose uptake observed in EHD2-deficient cells. Western blot analysis showed lowered insulin-stimulated phosphorylation of IRS-1 (Y612), Akt (S473), and AS160 (T642) at maximal insulin concentrations in EHD2 KO adipocytes compared with WT (Figure 2, A–D). Further, we found a reduction in submaximal insulin-stimulated phosphorylation of IRS-1 (Y612; Figure 2, A and D) and slightly reduced submaximal phosphorylation of Akt (S473, Figure 2B).

Protein expression levels of IRS-1, Akt, and AS160 in the basal state were similar comparing WT and EHD2 KO (Figure 2E and quantifications displayed as phospho/total protein levels for each target are shown in Supplemental Figure S1B). For comparison insulin signaling in WT and EHD2*-*deficient adipocytes in the chow-fed state is presented in Supplemental Figure S1C.

**FIGURE 3:**
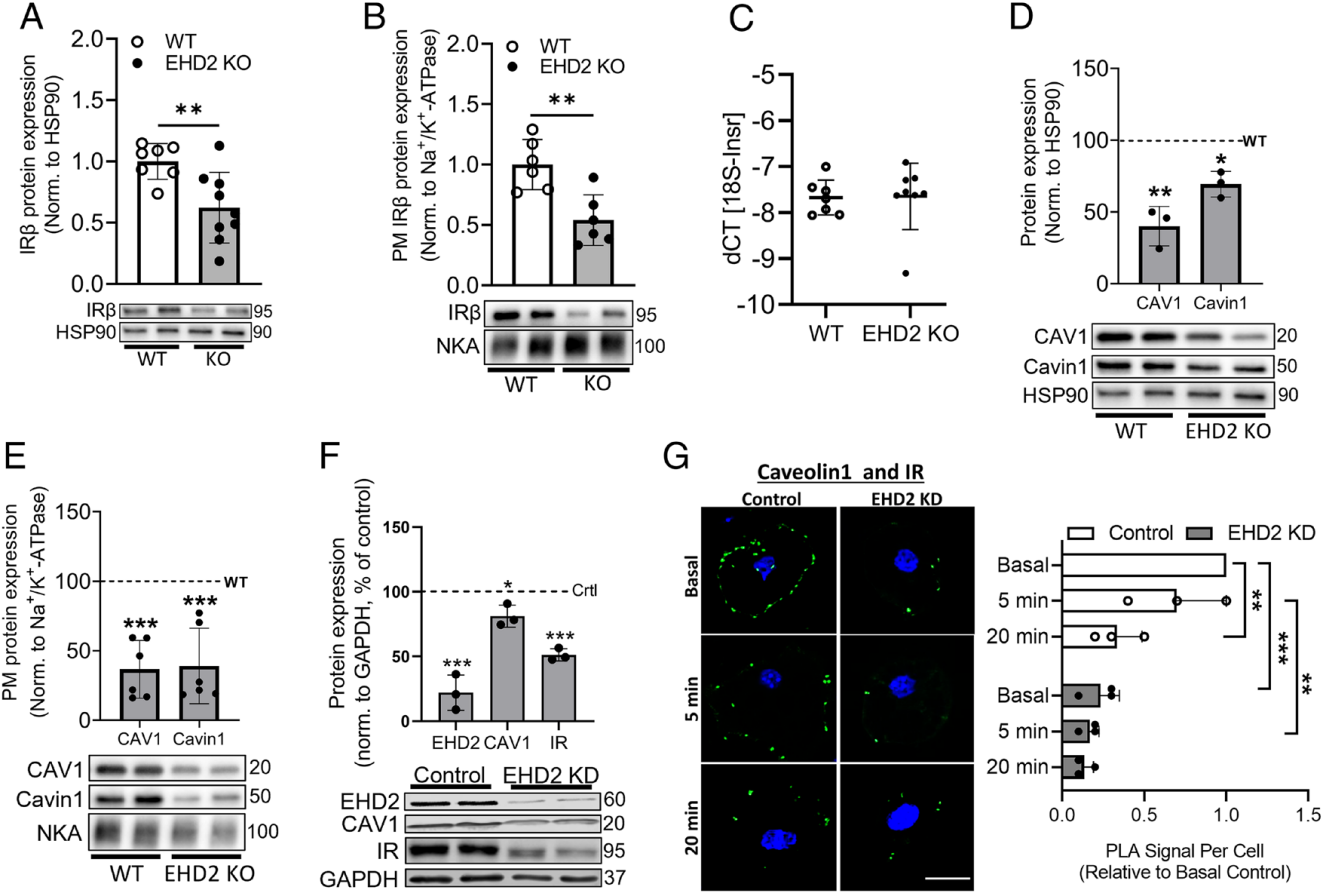
Impaired IR stability and blunted CAV1-IRβ interaction in EHD2 KO adipocytes. Protein levels of IRβ in (A) whole-cell and (B) PM. Data are displayed as mean ± SD and normalized to WT proteins levels. (A) *n* = 7 and 8 biological replicates and (B) *n* = 6 biological replicates/condition. (C) RNA was isolated from inguinal adipose tissue, and IR gene expression was examined using qPCR. Statistical analysis carried out on dCT values, and dCT was calculated as dCT = CT_ref_ - CT_goi_, *n* = 7 and 8 biological replicates. (D) Whole-cell and (E) PM CAV1 and cavin1 protein levels in primary adipocytes. Data are displayed as percentage of WT levels (WT = 100%, dashed line); *n* = 3 biological replicates/condition (D), *n* = 5 and 6 biological replicates (E). (F) Protein expression of EHD2, CAV1, and IRβ normalized to GAPDH in 3T3-L1 adipocytes of either control siRNA or EHD2 siRNA cells. Data are displayed as percentage of control levels (control = 100%, dashed line), *n* = 3 biological replicates. Statistical analysis was carried out using unpaired two-sample *t* test. Significance was determined according to *p ≤ 0.05, **p ≤ 0.01, ***p ≤ 0.001. (G) PLA was used to quantify the interaction of CAV1 and IRβ in 3T3-L1 adipocytes, fixed 96 h after gene silencing with control siRNA (Control) or EHD2 siRNA (EHD2 KD), and following 0 (Basal), 5 or 20 min stimulation with 100 nM insulin. Representative images, with PLA signal in green and nuclei staining (DAPI) in blue are shown alongside corresponding quantification of PLA signal per cell normalized to basal control. Mean ± SD of *n* = 3 independent experiments are shown. Statistical analysis was done using two-way ANOVA, Tukey’s Honest Significant Difference (TukeyHSD), *p < 0.05, **p < 0.01, ***p < 0.001. Scale bar = 10 µm. NKA = Na^+^/K^+^-ATPase.

As the IR is located and stabilized at caveolae and relies on caveolar integrity to function, we next assessed the expression of both IR and the core-caveolar proteins CAV1 and cavin1. We found a significant decrease (∼50%) in both total and PM-associated IRβ levels (Figure 3, A and B; see Supplemental Figure S1D for chow IRβ levels) in adipocytes from EHD2 KO mice compared with WT, whereas RT-qPCR analysis showed similar IR (*Insr*) mRNA expression (Figure 3C). Further, both CAV1 and cavin1 were significantly downregulated in both whole-cell lysates (∼60 and ∼40%, respectively, Figure 3D) and PM fraction (∼65%, Figure 3E) in EHD2 KO adipocytes compared with WT.

To address whether EHD2 KD affects IRβ and CAV1 interaction, we used PLAs to probe the interaction between these proteins in situ in siRNA-mediated *EHD2* gene-silenced (EHD2 KD) 3T3-L1 adipocytes. Consistent with our observations in primary adipocytes, EHD2 KD in 3T3-L1 cells reduced IRβ and CAV1 expression (∼50 and 20%, respectively; Figure 3F). PLA revealed a previously undescribed insulin-dependent reduction in the interaction between CAV1 and IRβ upon insulin stimulation in control cells (60% reduction 20 min after exposure to insulin), confirming and extending previous work identifying an important role for caveolae in IR signaling. Interestingly, we observed that the CAV1-IRβ interaction was significantly lower for EHD2 KD cells under all conditions tested, and insulin was without effect (Figure 3G). It should be noted that although the total cellular levels of these proteins are reduced in EHD2 KD adipocytes compared with control which may account for the reduced interactions in the absence of insulin, here we also compare interactions in response to insulin within each group that are independent of levels.

Together, these findings suggest that the lack of EHD2 alters caveolae integrity, which in turn negatively influences IRβ stability at the PM and downstream insulin signaling, ultimately reducing insulin-stimulated glucose uptake.

### Insulin-stimulated SNARE protein complex formation is impaired in EHD2-depleted 3T3-L1 adipocytes

To verify our findings in another adipocyte model and to examine whether our findings are related to EHD2 deficiency in adipocytes specifically (and not other factors related to whole-body EHD2 KO influencing adipocyte function), we made use of siRNA-mediated EHD2 gene silencing (EHD2 KD) in 3T3-L1 adipocytes.

**FIGURE 4:**
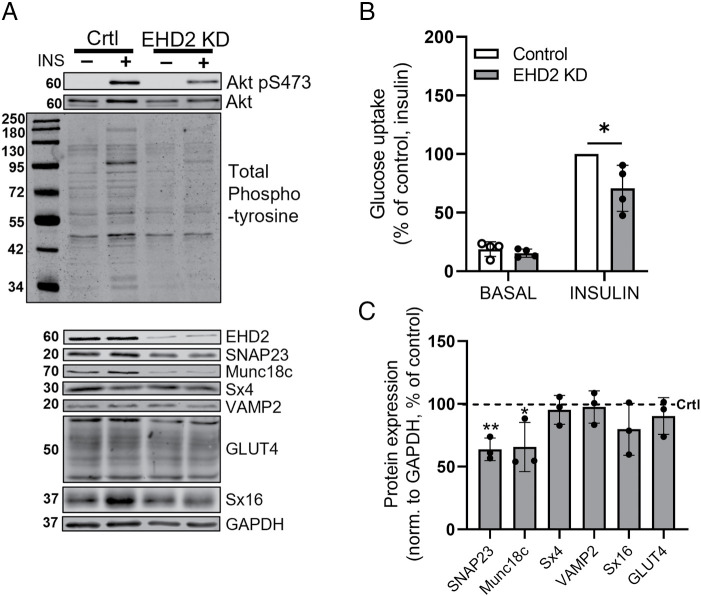
EHD2 KD in 3T3-L1 adipocytes is associated with impaired insulin signaling. (A) Representative Western blots of total phospho-tyrosine in 3T3-L1 adipocyte lysates collected 96 h after gene silencing with control siRNA or EHD2 siRNA, either untreated or following 20-min stimulation with 100 nM insulin (*n* = 2) and of protein levels of EHD2, SNARE proteins Syntaxin4 (Sx4), SNAP23, VAMP2, and Syntaxin16 (Sx16), as well as regulatory protein Munc18c, and GLUT4 (*n* = 3). GAPDH was used as a loading control and the calculations displayed in C were performed exclusively on basal samples. (B) Glucose uptake (2-deoxy-D-glucose) of 3T3-L1 adipocytes, 96 h after gene silencing with control siRNA (Control) or EHD2 siRNA (EHD2 KD), with (INS) or without (Basal) 20-min stimulation with 100 nM insulin. Data were corrected for nonspecific cellular isotope uptake by performing parallel assays in the presence of 10 μM cytochalasin B and normalized to those obtained in the insulin-stimulated control adipocytes for each data set. Mean ± SD of *n* = 4 independent experiments are shown. Statistical analysis was done using two-way ANOVA Tukey’s Honest Significant Difference (TukeyHSD), *p < 0.05. (C) Corresponding quantification of protein expression in EHD2 siRNA KD adipocytes in the absence of an acute insulin challenge (lanes labeled “-” in A) is normalized to GAPDH and expressed as a percentage of protein expression in control siRNA adipocytes. Mean and SD of *n* = 3 independent experiments are shown. Statistical analysis was conducted using unpaired two-sample *t* test, *p < 0.05, **p < 0.01.

Using a total-phosphotyrosine antibody, we found overall lowered insulin-induced tyrosine phosphorylation (Figure 4A) and impaired insulin-stimulated glucose uptake (Figure 4B) but similar GLUT4 levels (Figure 4C) in EHD2 KD cells consistent with findings described above in primary adipocytes. Because SNARE proteins underpin the fusion of GSV with the cell surface, we examined the expression of SNARE proteins in EHD2 KD cells. Both the t-SNARE SNAP23 and the SNARE regulatory protein Munc18c were significantly lower (∼40%) in EHD2 KD compared with control (Figure 4C), while total cellular expression of VAMP2, Sx4 and 16, SNARE proteins implicated in GLUT4 trafficking (Bryant *et al.*, 2002), remained unaltered (Figure 4C).

**FIGURE 5:**
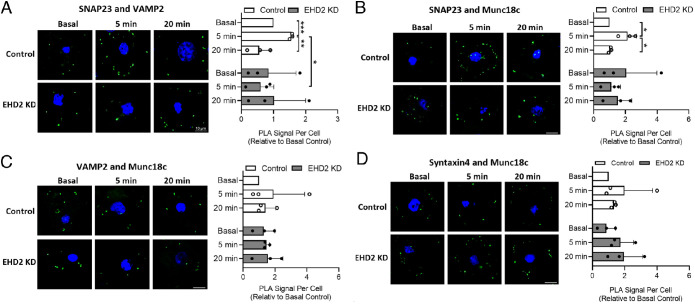
EHD2 KD in 3T3-L1 adipocytes causes impaired SNARE protein assembly. PLA was used to assess pairwise interactions of proteins in 3T3-L1 adipocytes, fixed 96 h after gene silencing with control siRNA (Control) or EHD2 siRNA (EHD2 KD), and following 0 (Basal), 5- or 20-min stimulation with 100 nM insulin. Representative images with PLA signal in green and nuclei staining (DAPI) in blue are shown alongside corresponding quantification of PLA signal per cell normalized to basal control for the following protein pairs: (A) SNAP23 and VAMP2; (B) SNAP23 and Munc18c; (C) VAMP2 and Munc18c; and (D) Syntaxin4 and Munc18c. Mean ± SD of *n* = 3 independent experiments are shown. Quantification was performed in ImageJ, and statistical analysis was done using two-way ANOVA, Tukey’s Honest Significant Difference (TukeyHSD), *p < 0.05, **p < 0.01, ***p < 0.001. Scale bar = 10 µm.

To address whether EHD2 KD affects the formation of the SNARE complexes required for GSV fusion, we used PLA to probe interactions between SNARE proteins in situ in control and EHD2 KD cells that were insulin-stimulated for 5 or 20 min or left untreated (Basal; Figure 5). We observed significant transient increases in insulin-stimulated interactions between SNAP23/VAMP2 and SNAP23/Munc18c in control cells (time point 5 min) consistent with previous studies (Kioumourtzoglou *et al.*, 2014). In marked contrast, these interactions were not affected by insulin in EHD2 KD cells (Figure 5). In line with previous reports (Kioumourtzoglou *et al.*, 2014), we found no effect of insulin on interactions between VAMP2/Munc18c or Sx4/Munc18c in either WT or EHD2 KD cells (Figure 5). These data underscore the importance of EHD2 to facilitate the formation of key SNARE complexes involved in insulin-stimulated GSV fusion events at the PM.

### EHD2 deficiency is associated with altered PM lipid composition

**FIGURE 6:**
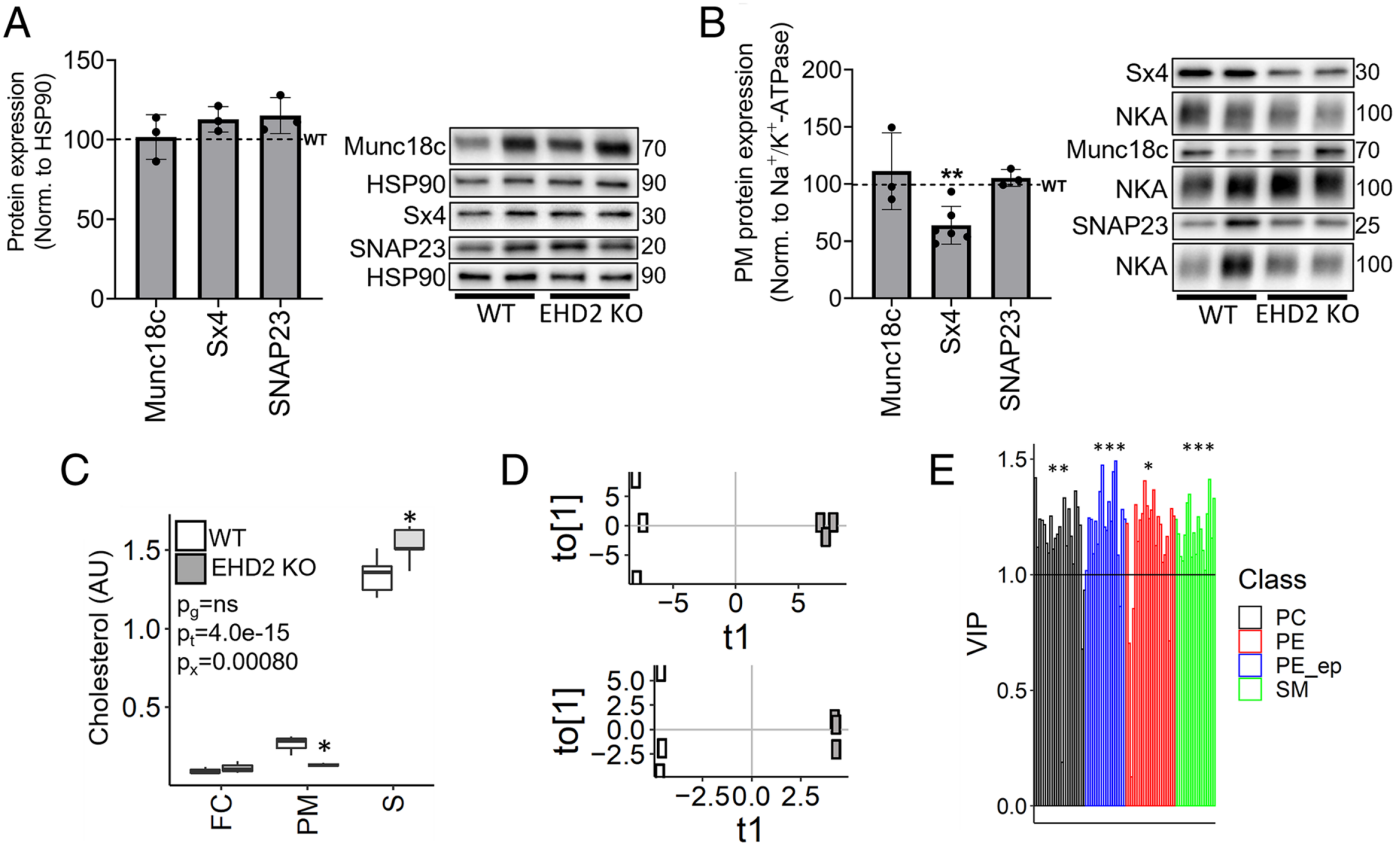
Reduced cholesterol and altered PM lipid content in EHD2 KO adipocytes. (A) Whole-cell and (B) PM samples were subjected to immunoblotting for Munc18c, Sx4, and SNAP23. All data are presented as percentage of WT levels (WT = 100%, dashed line), *n* = 3 biological replicates (A) and *n* = 3–6 (B). Data are displayed as mean ± SD, and unpaired two-sample *t* test was used for statistical analysis. Significance was determined according to **p ≤ 0.01. (C) Cholesterol levels in FC, PM, and serum (S) from WT and EHD2 KO mice. Differences assessed by two-way ANOVA, involving an interaction (x) between genotype (g) and sample (t) with a posthoc Student’s *t* test. (D) Score plots from OPLS-DA calculated on membrane lipidomic data acquired in positive (top) and negative (below) electrospray ionization mode. t1, first predictive component; to[1], first orthogonal component. WT in white, EHD2 in gray. (E) Lipids showing significant differences between WT and EHD2 KO PMs (variable importance of projection (VIP >1). PE, PE_ep, PC, and SM. Enrichment was accessed using *χ*^2^ statistics; PC_ep, *q* = 0.0013, PE, *q* = 0.011, PE_ep, *q* = 0.00081, SM, *q* = 0.00022. Significance was determined according to *p ≤ 0.05, **p ≤ 0.01 and ***p ≤ 0.001, *n* = 3 biological replicates. NKA = Na^+^/K^+^-ATPase. All displayed results were obtained from inguinal adipocytes 2 wk of HFD.

In an attempt to understand how EHD2 deficiency could impact SNARE complex formation, we turned back to primary adipocytes. Adipocytes from EHD2 KO did not exhibit altered total levels of Sx4 (or SNAP23 and Munc18c; Figure 6A). However, Sx4 levels were strikingly reduced (∼40%) in the PM-enriched fraction of EHD2 KO adipocytes (Figure 6B).

As the structure of individual SNARE proteins (Saito *et al.*, 2012; Fezoua-Boubegtiten *et al.*, 2019; Lakomek *et al.*, 2019; Wang *et al.*, 2020), their localization to specific PM domains (Chamberlain *et al.*, 2001; Lang *et al.*, 2001; Salaun *et al.*, 2005), and the process of SNARE protein complex formation as well as GLUT functionality (Hresko *et al.*, 2016) is influenced by lipid environment, we next performed lipidomic analysis of isolated primary adipocytes to resolve if differences in PM composition could contribute to the lowered insulin-induced effects in EHD2 KO adipocytes. Consistent with this idea, we observed lower levels of cholesterol in the PM fraction of EHD2 KO adipocytes, whereas no differences were observed in the isolated FC fraction (Figure 6C). This alteration does not reflect a global lipid remodulation, as serum levels of cholesterol were higher in the EHD2 KO mice (Figure 6C). One possible explanation of this observation stems from work implicating Sx6 in the delivery of microdomain-associated lipids and proteins to the cell surface. Sx6 is the Q_b,c_ SNARE syntaxin that forms a functional tSNARE with the Q_a_ SNARE Sx16 (Wang *et al.*, 2017) and plays a key role in sorting GLUT4 through the endosomal system. Disruption of Sx6 impairs delivery of caveolar proteins to the cell surface (Choudhury *et al.*, 2006), indicating that cholesterol, caveolae, and Sx6/16 are functionally linked. Mass spectromery analysis of proteins coimmunoprecipiated with Sx16 identified EHD2 as an associated protein (Table 1). Analysis of these data revealed that it was the major protein in the immunoprecipitated sample, and peptides corresponding to 77% of the EHD2 protein were identified. Importantly, EHD2 was more abundant in these immunoprecipitates than mVps45, the Sec1/Munc18 binding partner of Sx16 (Table 1).

**TABLE 1:** Summary of mass spectrometry data of Sx16 coimmunoprecipitation analysis.

Accession #	Name	Score	Coverage	# Unique peptides	# Peptides	#PSMs
IPI00402968.1	EHD2	2285.04	76.98%	41	42	88
IPI00124291.1	mVps45	1230.21	56.32%	34	34	47

Sx16 was immunoprecipitated from 3T3-L1 adipocytes incubated in serum-free media for 2 h prior to lysis (i.e., in the basal state). Sx16 was immunoprecipitated and processed for mass spec analysis as outlined in the method section. Shown are data for EHD2 and mVps45. EHD2 was the most abundant protein identified in this experiment. Score is the sum of ion scores of all peptides that were identified. Coverage is the percentage of the protein sequence covered by identified peptides. #PSM refers to the number of peptide spectrum matches. #Unique peptides is the number of peptides common to a protein group and which do not occur in the proteins of any other group. #Peptides is the total number of distinct-peptide sequences identified in the protein group. #PSM is the number of peptide spectrum matches.

With respect to the lipid profile, PCA revealed a systematic difference in the membrane fraction and serum between the EHD2 KO mice and WT, but not so for the FC (Supplemental Figure S2). These changes were investigated in more detail using OPLS-DA, revealing a systematic difference in PM lipidome between WT and EHD2 KO mice (Figure 6, D and E). Notably, an enrichment of PE, PE ether lipids (PE_ep), phosphatidylcholine (PC), and SM were found among significantly altered lipids, all of which showed lower levels in adipocyte PMs from EHD2 KO mice. No systematic difference was observed in the FC and in the serum lipidomes. Possibly, these differences in membrane lipid composition are related to the changes in insulin-mediated SNARE complex formation, IR stability, and GLUT4 integrity and vesicle fusion.

## DISCUSSION

We previously confirmed a central role of EHD2 to sustain lipid metabolism (Fryklund *et al.*, 2021). In the latter study, we found that insulin-induced inhibition of adipocyte lipolysis was diminished in EHD2 KO mice (Fryklund *et al.*, 2021), and therefore we set out to explore whether other insulin-regulated cellular processes were affected in EHD2-deficient cells. Further, we could also elucidate in the respective study that 2 wk of HFD serves as a time-point at which we can observe similar adipocyte size in EHD2 KO and WT adipocytes, excluding that the observed differences are due to changes in adipocyte size (Fryklund *et al.*, 2021).

In this study, we demonstrate that both basal- and insulin-stimulated glucose uptake are impaired in EHD2 KO adipocytes after short-term HFD feeding. These findings are in line with previous findings from Park *et al.*, reporting that inhibition of EHD2 (using an EHD2-specific antibody) is associated with impaired insulin-stimulated GLUT4 translocation in primary rat adipocytes (Park *et al.*, 2004). Consistent with this, we observed reduced insulin-stimulated glucose uptake and GLUT4 translocation in EHD2 KO adipocytes compared with WT controls (Figure 1). The fact that we see comparable differences in both glucose tracer uptake assays using either a metabolized or a nonmetabolized glucose tracer suggests that the differences in glucose uptake originate from impaired glucose transport rather than metabolism (Figure 1). The differences in basal glucose uptake could be linked to the reduced number of GSVs in EHD2 KO adipocytes, which could consequently lead to altered GLUT4 cycling. Our results demonstrate similar GLUT4 expression and similar basal GLUT4 in PM in both genotypes (EHD2 KO and WT; Figure 1, E, G, and I) and thus suggest that mechanisms other than those related to GLUT4 levels contribute to diminished glucose uptake. Rather, we observed a decreased magnitude of GLUT4 translocation in EHD2 KD adipocytes, prompting us to consider the mechanisms which may underlie this.

We observed significantly impaired phosphorylation of key insulin signaling mediators IRS-1 (Y612), Akt (S473), and AS160 (Thr642; Figure 2), which may contribute to the observed decrease in insulin-stimulated glucose uptake in EHD2 KO adipocytes. These findings were replicated using siRNA-mediated EHD2 KD in 3T3-L1 cells, which also resulted in reduced insulin signal transduction and lowered insulin-stimulated glucose uptake (Figure 4). However, proximal insulin signaling holds a high spareness, meaning that reduced phosphorylation of IRS-1 and Akt can still induce maximal biological response to insulin. Therefore maintained glucose uptake could be observed despite impaired proximal insulin signaling (Frank *et al.*, 1981; James *et al.*, 2021).

In search for the cause behind impaired insulin signaling, we found significantly reduced protein levels of IRβ, CAV1, and cavin1 (total and PM-localized) in EHD2 KO adipocytes, without reduced IR mRNA levels (Figures 3). These observations suggest that EHD2 deficiency negatively affects IRβ stability. In support of this, Cohen *et al.* showed that CAV1 deficiency evokes proteosomal degradation of the IR (Cohen *et al.*, 2003), and CAV1 KD is associated with reduced stability and diminished expression of IRβ and GLUT4 in 3T3-L1 adipocytes (Gonzalez-Munoz *et al.*, 2009). Further, data from Nystrom *et al.* (1999) elucidated that IRβ contains a CAV1-specific binding motif; upon CAV1 KO this binding was disrupted, resulting in markedly decreased cell surface IR expression and a defect in IRβ autophosphorylation. Consistent with this, Yamamoto *et al.* (1998) demonstrated that caveolin controls IRβ kinase activity in 293T cells. Using PLA, we revealed an interaction between CAV1 and IRβ in control cells, which significantly decreased in response to insulin (Figure 3). This is in line with the notion that caveolae-associated IRs are a key focal point of insulin signaling, and that IRβ levels in caveolae drop in response to insulin treatment, possibly reflecting the subsequent ligand-induced internalization of the receptor which is known to be necessary to propagate insulin signaling (Hall *et al.*, 2020). Interestingly, CAV1-IRβ interaction was significantly reduced in EHD2 KD cells in the basal state and was not sensitive to insulin, in contrast to control cells. While the IR was shown to both localize and function in caveolae (Gustavsson *et al.*, 1999), Foti *et al.* (2007) demonstrated that the IR is specifically enriched at the neck of caveolae, suggesting that EHD2 and the IR are found in close proximity in caveolae. Our data indicate that EHD2 is necessary to sustain IR downstream signaling, probably mediated through a caveolae-dependent IR stabilization, which preserves IR autophosphorylation.

Mattheus *et al.* (2020) could previously demonstrate that the amount of membrane-detached caveolae is increased in white adipose tissue from EHD2 KO mice. Accordingly, we observed irregular caveolae shape and size in EHD2-deficient primary adipocytes in our previous study (Fryklund *et al.*, 2021). The significantly decreased content of caveolar core proteins CAV1 and cavin1 in the PM, presented in the current study (Figure 3) is consistent with hypothesis that caveolae integrity is potentially altered in EHD2-deficient cells. In support of this, we observed lowered cholesterol and decreased levels of SM, PE, PC in the PM from EHD2 KO adipocytes, indicating that a large share of caveolae-characterizing lipids is less abundant in the PM of these adipocytes. These observations are supported by studies that have linked Sx6/Sx16 function with the delivery of cholesterol and cavaeolae components to the cell surface (Choudhury *et al.*, 2006; Wang *et al.*, 2017). Here we show that EHD2 interacts directly with Sx16 (Table 1), and thus identify a potential link between EHD2 function and both caveolar integrity and insulin signaling. This is supported by studies using Methyl-β-cyclodextrin-induced caveolae depletion that revealed a causal link between caveolae integrity, insulin signal transduction, and glucose uptake (Parpal *et al.*, 2001; Karlsson *et al.*, 2004). Herein, we demonstrate that IR stability and downstream signaling events are impaired concurrently with altered PM lipid and protein profile in EHD2-deficient adipocytes. This is firmly in line with previous reports showing a strong relation between IRβ signaling and PM lipid environment, including caveolae-lipid microenvironment, as a controlling agent of IRβ function and downstream signaling (Yamamoto *et al.*, 1998; Foti *et al.*, 2007; Perona, 2017; Suresh *et al.*, 2021). We further extend these observations to suggest a novel interaction between EHD2 and Sx16 may underpin these observations, but further studies are required to define these observations mechanistically.

It is well established that not only the IR but also the localization, assembly, and function of SNARE proteins as well as GLUT integrity is influenced by the membrane lipid composition. Proteomic studies have shown an enrichment of SNAP isoforms and the vesicle SNARE protein VAMP2 in isolated caveolae fractions (Schnitzer *et al.*, 1995; Matthaeus and Taraska, 2020) and cholesterol levels are known to influence the function of key SNARE proteins involved in insulin-stimulated GLUT4 translocation to the cell surface (Chamberlain and Gould, 2002). Additionally, it has been postulated that membrane lipid remodulation is initiated by protein-dependent formation of membrane heterogeneities, which then are stabilized by recruitment of lipids (Harayama and Riezman, 2018). Hence, an altered expression of EHD2 may promote changes in lipid rafts or other PM cholesterol-containing domains, perhaps by Sx16-dependent mechanisms. Notably, among the lipids showing reduced PM levels in EHD2 KO, we found the bilayer-disrupting lipid classes PE and the related ether lipids (PE_ep; Figure 6), which are crucial mediators of fusion and fission events (Harayama and Riezman, 2018). Moreover, levels of both cholesterol and SM lipids were reduced (Figure 6), emphasizing their coexistence in membrane nanodomains (Sezgin *et al.*, 2017). Importantly, Hresko *et al.* (2016) demonstrated that PE, PC, and Cholesterol (lipids we also identified as crucially reduced in EHD2 KO PMs) are among the membrane lipids that exert the most critical effects on GLUT4 transport capacity and GLUT4 stabilization, possibly explaining the herein observed differences in glucose uptake.

The differences in PM cholesterol and lipid composition detected in EHD2-depleted cells may also contribute to the impaired SNARE protein assembly revealed from our PLA analysis (Figure 5). Our previous studies identified an insulin-dependent increase in SNARE complex formation, revealed by increases in PLA signal between SNAP23 and VAMP2, and SNAP23 and Munc18c 5 min after exposure to insulin (Kioumourtzoglou *et al.*, 2014); a result recapitulated here (Figure 5). In marked contrast, EHD2 KD cells did not exhibit these insulin-dependent changes, despite broadly similar PLA signals in the absence of insulin. One interpretation of these data is that EHD2 KD results in impairment of early events (5 min after exposure to insulin) in GLUT4 trafficking. We and others have reported a cholesterol-dependent clustering of the SNARE proteins involved in GLUT4 vesicle fusion with the PM (Lang *et al.*, 2001; Chamberlain and Gould, 2002), hence the impairment in SNARE function in EHD2 KD cells might explain the observed reduction in insulin-stimulated glucose transport (Figure 4). The fact that we found reduced expression of Sx4 in the PM, but not total cell lysates, in EHD2 KO inguinal adipocytes (Figure 6) further emphasizes that SNARE distribution is impaired in EHD2 KO adipocytes, which indeed could contribute to impaired GLUT4 exocytosis and hence lowered glucose uptake.

In conclusion, we present data that emphasize the importance of caveolae integrity for insulin signaling and glucose uptake. Further, our data suggest that EHD2 deficiency negatively influences IRβ stability, CAV1-IRβ interaction, insulin signaling, and downstream events including SNARE protein complex formation and GSV fusion. We hypothesize these alterations are caused by impaired caveolae integrity and altered membrane lipid composition, which in turn influences IR stability and membrane-trafficking events.

## MATERIALS AND METHODS

Request a protocol through *Bio-protocol*.

### Materials

IRS-1 (#06-248, RRID:AB_2127890, Merck Millipore, Billerica, USA), phospho IRS-1 Y612 (#44-816G, RRID:AB_2533768, Life Technologies, Carlsbad, USA), AS160 Akt substrate of 160 kDa (#07-741, RRID:AB_492639, EMD Millipore, Darmstadt, Germany). Protein kinase B (Akt; #4691, RRID:AB_915783), phospho Akt S473 (#4060S, RRID:AB_2315049), phospho AS160 Thr642 (#4288S, RRID:AB_10545274), glyceraldehyde 3-phosphate dehydrogenase (GAPDH; RRID:AB_2536381, Thermo Fisher Scientific Catalogue, Massachusetts, USA) and sodium-potassium ATPase (Na^+^/K^+^-ATPase; #96124, RRID:AB_2800256) were from Cell Signaling (Cambridge, UK). CAV1 (#610407, RRID:AB_397789) and Heat shock protein (HSP) 90 antibody (#610418, RRID:AB_397798) were from BD Biosciences (Franklin Lakes, USA). Cavin 1 (#ab48824, RRID:AB_882224) and EHD2 (Abcam catalogue# ab154784, RRID:AB_2927498) were from Abcam (Cambridge, UK). Mammalian uncoordinated-18c (Munc18c; #116 202, RRID:AB_2619785), Syntaxin 4 (Sx4; #110 042, RRID:AB_887853), vesicle-associated membrane protein 2 (VAMP2; #104 403, RRID:AB_2864782), synaptosome associated protein 23 (SNAP23; #111 213, RRID:AB_10805651), and Syntaxin 16 (Sx16; #110 162, RRID:AB_887799) were all from Synaptic Systems (Coventry, UK). GLUT4 used for 3T3-L1 analysis (#PA1-1065 RRID:AB_2191454, Thermo Fisher Scientific Catalogue, Massachusetts, USA), insulin-regulated aminopeptidase (IRAP; #sc-365300, RRID:AB_10844333) and IRβ (#sc-711, RRID:AB_631835) were from Santa Cruz Biotechnology (Dallas, US). Phosphotyrosine antibody, clone 4G10 (EMD Millipore, Darmstadt, Germany, RRID:AB_2891016). IRAP antibodies used with 3T3-L1 cells were kindly provided by Paul Pilch (Boston University, USA) and GLUT4 for primary adipocytes was kindly provided by Sam Cushman, α-Tubulin (#T9026, RRID:AB_477593, Sigma-Aldrich, St. Louis, USA), Syntaxin-16 (Synaptic System #110 161; RRID:AB_2198368), and fluorescence-conjugated (Alexa Fluor) secondary antibodies and BODIPY 493/503 (D3922) were purchased from (Molecular Probes, Waltham, USA).

### Animals and diet intervention

C57BL6/N mice with global deletion of exon 3 of the *Ehd2* gene (referred to as EHD2 KO or KO in all figures) as described (Matthaeus *et al.*, 2020) were kindly provided by Oliver Daumke (MDC, Germany). Genotyping was carried out to confirm the deletion of the *EHD2* gene*.* EHD2 KO and corresponding control wild-type (WT) C57BL6/N mice were bred in parallel to generate sufficient number of animals for cellular analysis. Animals were kept on a 12-h light cycle with nonrestricted food and water supply and were acclimatized 1 wk before starting the intervention. Animals (*n* = 2–5/cage) were fed either chow or HFD (#D12492, 60E % fat, Research Diets, New Brunswick, USA), for 2 wk. At the time of termination, inguinal adipose tissues were excised, weighed, and used for adipocyte isolation. Data were obtained from male mice at ∼12 wk of age. Terminal serum samples were collected from *vena saphena* for serum cholesterol analysis. All animal procedures were approved by the Malmö/Lund Committee for Animal Experiment Ethics, Lund, Sweden.

### Isolation of primary adipocytes

Primary adipocytes were isolated from inguinal adipose tissue depot as described previously (Rodbell, 1964). The cells were suspended in Krebs Ringer Bicarbonate HEPES (KRBH) buffer, pH 7.4, containing 200 nM adenosine, and 3% (wt/vol) bovine serum albumin (BSA).

### Glucose uptake in primary adipocytes

Glucose uptake in primary adipocytes was determined as previously described (Gliemann *et al.*, 1984). Briefly, cells (7.5% [vol/vol] suspension) were incubated with or without insulin (concentrations shown in Figure Legends) in KRBH buffer in triplicates for 30 min, followed by the addition of D-^14^C(U)-glucose (2.5 µl/ml, NEC042, Perkin Elmer, Waltham, USA), and an additional 30 min of incubation. The uptake was terminated by spinning 300 µl of each cell suspension in microtubes containing 80 µl dinonylphtalate oil. The cell fraction was collected, dissolved in scintillation fluid (Optima Gold, Perkin Elmer), and subjected to scintillation counting. Glucose uptake in 3T3-L1 adipocytes was performed as outlined (Roccisana *et al.*, 2013).

### Western blot analysis

For Western blot analysis of basal and insulin stimulated adipocytes, cells were washed with KRBH buffer without BSA before being lysed in lysis buffer containing 50 mM Tris/HCl pH 7.5, 1 mM EGTA, 1 mM EDTA, 0.27 M sucrose, 1% NP-40, and complete protease and phosphatase inhibitor cocktail (Roche, Basel, Switzerland). Cell lysates were centrifuged for 10 min at 13,000 × *g* and protein concentrations were determined using the Bradford method (Bradford, 1976). Samples were subjected to PAGE and electro-transfer to nitrocellulose membranes. Membranes were blocked with nonfat dry milk (5% [wt/vol]) and probed with the indicated antibodies. Detection was performed using horseradish peroxidase-conjugated secondary antibodies and enhanced chemiluminescence reagent. The signal was visualized using a Bio-Rad Image camera (Bio-Rad, Hercules, USA). HSP90, α-tubulin, or Na^+^/K^+^-ATPase were used throughout as loading controls.

### TIRF imaging

For TIRF imaging, we used a commercial TIRF system based on a Nikon Ti-E eclipse microscope equipped with a 100 × Apo TIRF DIC oil immersion objective NA of 1.49 (Nikon Instruments), an iXon Ultra DU-897 EMCCD camera (Andor Technology), and four main lasers, 405 nm (Cube, Coherent), 488 nm (Melles-Griot), 561 nm (Sapphire, Coherent), and 640 nm (Cube, Coherent) with corresponding filter sets. Isolated cells were fixed using 4% paraformaldehyde and labeled with antibodies in a buffer containing 1% BSA, 1% goat serum, and 0.05% saponin, 1 h per labeled antibody, and imaged as previously described (Wasserstrom *et al.*, 2018). Puncta detection was performed to identify GSVs using Blob Finder (ZEISS Arivis software) at size cutoff set at <140 nm stepwise for each channel. The number of GSVs was determined by detection of puncta co-labelled with GLUT4 and IRAP. Signals detected within 40 nm distance were considered to arise from the same puncta.

### RNA-sequencing analysis

RNA-sequencing analysis data originate from Hansson *et al.*, 2018. Data analysis was performed accordingly.

### RT-qPCR

Total RNA was extracted using miRNeasy mini kit (Qiagen #74104). RNA purity and concentration was assessed using a Nanodrop spectrophotometer (Thermo Fisher Scientific). RT-qPCR analysis was performed using the Quantifast SYBR Green RT-PCR kit (Qiagen #204156) and Quantitect primer assays for *18S* (QT02448075) and *Insr_1* (QT00287903). Primer sequences are considered proprietary information by Qiagen. mRNA expression levels were measured using a StepOnePlus real-time thermal cycler (Applied Biosystems Waltham, United States) and displayed as dCT = CT_ref_ – CT_goi_. 18S rRNA was used for normalization (CT_ref_).

### PM fractionation

PM fractions for determination of insulin-stimulated GLUT4 translocation were prepared using a method described by (Nishiumi and Ashida, 2007). Briefly, isolated adipocytes were incubated with our without insulin (1 nM) for 30 min. Subsequently, cells were washed with KRBH without BSA twice and lysed in a buffer containing 50 mM Tris/HCl pH 8.0, 0.5 mM DTT, 0.1% NP-40 and additional protease and phosphatase inhibitors (complete Ultra, Phospho-Stop, Roche, Basel, Switzerland). Cell lysates were centrifuged at 1000 × *g*, 10 min, 4°C. The fat layer was removed and the pellet was washed in 50 mM Tris/HCl pH 8.0, 0.5 mM DTT, and additional protease and phosphatase inhibitors. The supernatant was discarded, and the pellet was dissolved in 50 mM Tris/HCl pH 8.0, 0.5 mM DTT, 1 % NP-40, and additional protease and phosphatase inhibitors. After 1 h on top of ice with occasional shaking, the dissolved pellet was centrifuged at 16,000 × *g*, 20 min, 4°C, and the supernatant was collected as the PM fraction. PM fractions were subjected to subsequent Western blot analysis.

For assessment of IRβ, CAV1, cavin1, and SNARE protein levels as well as lipidomic analysis, PM fractions were obtained in a detergent-free way according to a protocol by (Gustavsson *et al.*, 1996). Briefly, primary adipocytes were lysed in buffer containing 10 mM Tris/HCl, pH 7.4, 1 mM EDTA, and protease inhibitors (complete Ultra, Phospho-Stop, Roche, Basel, Switzerland) using a dounce homogenizer (all steps were carried out at 0–4°C). Cell debris and nuclei were removed by centrifugation at 400 × *g* for 10 min. A PM-containing pellet was obtained by centrifugation at 16,000 × *g* for 20 min and was resuspended in 10 mM Tris/HCl, pH 7.4, 1 mM EDTA, and protease inhibitors (complete Ultra, Phospho-Stop, Roche, Basel, Switzerland). Purified PMs were obtained by sucrose density gradient centrifugation. Accordingly, the PM containing suspension was layered on a 1.12 M sucrose cushion and centrifuged for 60 min at 140,000 × *g*. PM fractions were collected and centrifuged at 170,000 × *g* for 20 min, the supernatant was discarded, and the pellet was used as a the adipocyte PM fraction for subsequent analysis.

### Cell culture

3T3-L1 fibroblasts, purchased from the American Tissue Culture Collection (RRID:CVCL_0123), were grown, maintained, and differentiated into mature adipocytes as outlined previously (Roccisana *et al.*, 2013; Sadler *et al.*, 2015). siRNA-mediated geneKD in 3T3-L1 adipocytes was performed as previously described (Duan *et al.*, 2022). EHD2 (Thermo Fisher Scientific, MSS281816) or scrambled siRNA (SCR; Ambion 4390844) was added to 1.2 ml Opti-MEM to a final concentration of 40 nM with 48 μl TransIT-X2 transfection reagent (Mirus Bio). At 6 d postdifferentiation, 3T3-L1 adipocytes grown on a 10-cm dish were washed once with phosphate-buffered saline (PBS), trypsinized with 2 ml Life Technologies TrypLE Express enzyme, resuspended in DMEM/FBS medium, and centrifuged at 200 × *g* for 5 min. The supernatant was removed, and pelleted cells were resuspended in 13.5 ml DMEM/FBS medium. Nine-hundred microliter cell suspension and 100 µl EHD2 or SCR siRNA/TransIT-X2/Opti-MEM mixture were reseeded onto each well of a 12-well plate. For proximity ligation assays (PLA), these wells contained 13-mm glass coverslips. Cells were used in experiments 4 d following siRNA KD. Cells were serum starved 2 h before all experiments.

### Immunoprecipiation and proteomics

Sx16 was immunoprecipitated from lysates of d 10 postdifferentiation 3T3-L1 adipocytes prepared as described (Bremner *et al.*, 2022). Two micrograms of anti-Sx16 (Synaptic System #110 161; RRID:AB_2198368) was used in immunoprecipitation from 1 mg of adipocyte lysate and the immunoprecipitated material separated on SDS–PAGE. All subsequent steps were performed at the FingerPrints Facility, University of Dundee. Samples were washed, then reduced/alkylated with DTT/IAA, respectively, and then digested overnight (16 h) with trypsin (Modified Sequencing Grade, Roche, UK). Peptides were extracted and dried in a SpeedVac, resuspended in 1% formic acid, centrifuged and analyzed using an Ultimate 3000 RSLCnano system (Thermo Fisher Scientific). Samples were injected and washed on C18 trap with 0.1% formic acid and after 3-min wash gradient formed with 0.1% formic acid and 80% acetonitrile on 0.08% formic acid. Peptides were initially trapped on an Acclaim PepMap 100 (C18, 100 µm × 2 cm, Thermo Fisher Scientific) and then separated on an Easy-Spray PepMap RSLC C18 column (75 μm × 50 cm, Thermo Fisher Scientific). Samples were transferred to LTQ OrbiTrap Velos Pro mass spectrometer via an Easy-Spray source with temperature set to 50°C and a source voltage of 2.05 kV. OrbiTrap Velos Pro RAW files were analyzed with Proteome Discoverer (Version 1.4) using Mascot (Version 2.4) as a search engine. IPImouse was used as a database.

### PLA

Following siRNA KD as previously described, 3T3-L1 adipocytes were reseeded onto 13-mm coverslips in 12-well plates. Following stimulation with 100 nM insulin for 5 or 20 min as indicated, cells were washed three times with PBS and fixed for 20 min with 1-ml warm 4% wt/vol paraformaldehyde. Cells were then washed three times with PBS and incubated with 1-ml quenching buffer (50 mM NH_4_Cl in PBS) for 10 min followed by a further three washes with PBS. Cells were incubated with 1 ml permeabilization buffer (0.1% vol/vol Triton X-100 in PBS) for 5 min. PLA on fixed and permeabilized 3T3-L1 adipocytes was carried out using the NaveniFlex MR kit (Navinci NF.MR.100) according to the manufacturer’s instructions. Briefly, cells were blocked to prevent nonspecific binding of antibodies before being incubated with a pair of primary antibodies from mouse and rabbit host species, respectively; Sx4 (RRID:AB_887853)/Munc18c (#H00006814-B01P, RRID:AB_2302682, Novus Biologicals, Centennial, US); CAV1 (#ab2910, RRID:AB_303405)/IRβ (#ab69508, RRID:AB_1209215, Abcam); VAMP2 (#104211, RRID:AB_887811, Synaptic Systems)/Munc18c(ab224625);SNAP23 (RRID:AB_10805651)/VAMP2 (RRID:AB_887811); SNAP23 (RRID:AB_10805651)/Munc18c (RRID:AB_2302682). Cells were then incubated with a pair of anti-mouse and anti-rabbit navenibodies before undergoing a series of enzymatic reactions before the application of the detection fluorophore. Following the NaveniFlex PLA protocol, cells were incubated with 1 μg/ml 4′,6-diamidino-2-phenylindol (DAPI) to stain DNA. All incubations took place in a humidity chamber at 37°C and cells were washed with TBST (20 mM Tris/HCl, 150 mM NaCl, 0.05% [vol/vol] Tween) between each step. All reaction volumes were 40 µl per 13-mm coverslip. Signals were visualized using a Leica TCS SP8 system with a 63× oil immersion objective. For quantification, signals in 50–70 imaged cells per experimental condition were counted in ImageJ, using the protocol and parameters previously described (Lopez-Cano *et al.*, 2019). Statistical analyses were done by two-way ANOVA testing. Figures and plots are representative of results from three independent experiments.

### Lipidomics

Detergent-free PM fractions were prepared as described above. Lipids were extracted and samples prepared as previously elucidated in detail (Herzog *et al.*, 2020), with the only exception being the fat cake (FC), which was evaporated and directly dissolved in isopropanol/water (9/1, vol/vol). Lipidomic analyses were performed on an Agilent 1290 Infinity UHPLC system coupled to an Agilent 6495 QqQ-MS (Agilent Technologies, Santa Clara, CA) operated in dynamic multiple-reaction-monitoring (MRM) mode. Separation of lipids was performed as previously described (Herzog *et al.*, 2020). The mass spectrometer was operated in positive and negative electrospray ionization mode (ESI^+^ and ESI^–^, respectively) with a fragmentor voltage of 380 V, a cell acceleration voltage of 5 V, a gas temperature of 200°C, a gas flow rate of 14 l/min, a nebulizer pressure of 20 psi, a sheath gas temperature of 250°C, and a sheath gas flow-rate of 11 l/min. The capillary voltage was 3000 V for ESI^+^ and 2500 V for ESI^−^, the high/low pressure iFunnel RF was 200/100 V and 90/60 V for ESI^+^ and ESI^−^, respectively, and the nozzle voltage 1500 V for both modes. MRM transitions were based on (Takeda *et al.*, 2018) and are given in Supplemental Table S1 (ESI^+^) and Supplemental Table S2 (ESI^−^). Data were processed using MassHunter Qualitative Analysis and Quantitative Analysis (Agilent Technologies, Santa Clara, CA). Cholesterol levels were measured in PM, FC, and serum samples collected from WT and EHD2 KO mice (Fryklund *et al.*, 2021) by gas chromatography mass spectrometry, as previously described in detail (Danielsson *et al.*, 2010).

### Statistical analyses

Statistical analyses were carried out as indicated in each figure legend using GraphPad Prism 9 (GraphPad Software) software. PLA data were analyzed using two-way ANOVA testing performed in R using RStudio. Significance was determined according to * *p* ≤ 0.05, ** *p* ≤ 0.01, *** *p* ≤ 0.001, and **** *p* ≤ 0.0001. All data are displayed as mean ± SD. Lipidomic data were analyzed in R 3.6.1 using ANOVA (aov), principal component analysis (PCA; prcomp), the Student’s *t* test (t.test) and the Chi-square test (chisq.test) from the stats package. Orthogonal projections to latent structures discriminant analysis (OPLS-DA) was conducted using opls (ropls package). The *p* values are reported as q values after adjustment for multiple testing using the Benjamini-Hochberg method (p.adjust, stats). Data were illustrated using ggplot (ggplot2).

### SUMMARY

EH domain-containing protein 2 (EHD2) is one of the highest upregulated genes at the early stage of adipose tissue expansion. Using knockout mice and cell models, we show that EHD2 plays a key role in proximal and distal insulin signaling, and is important for maintenance of the plasma membrane milieu and insulin receptor stability in adipocytes.

## Abbreviations used:

AS160: Akt substrate of 160 kD
CAV1: Caveolin-1
EHD2: EH domain-containing protein 2
KD: Knockdown
KO: Knockout
GSV: GLUT4 storage vesicles
GLUT4: Glucose transporter 4
HFD: High-fat diet
HSP90: Heat shock protein 90
IRβ: Insulin receptor beta
IRS-1: Insulin receptor substrate-1
NKA: Na ^+^/K ^+^-ATPase
PKB (Akt): Protein kinase B
PLA: Proximity ligation assay
PM: Plasma membrane
SNARE: Soluble N-ethylmaleimide-sensitive factor-attachment protein receptors
