# Supplementary material for: EHD2 regulates plasma membrane integrity and downstream insulin receptor signaling events
Source: Mol Biol Cell. 2023 Oct 31;34(12):ar124. doi: 10.1091/mbc.E23-03-0078 (PMC10846623; doi:10.1091/mbc.E23-03-0078)

Supplemental Materials

Molecular Biology of the Cell

Neuhaus *et al*.

Supplementary Figure S1. Insulin signaling in WT and EHD2 KO adipocytes from chow-fed mice
(A) Insulin stimulated IRAP translocation (1nM, 30 min) (left) and non-stimulated levels of IRAP in the PM (right) of inguinal adipocytes from HFD mice. (B) Non-stimulated (basal) protein levels of IRS-1, Akt and AS160 and pIRS Y612, Akt S473 and AS160 Thr642 displayed as phosphorylated over total protein level in WT and EHD2 KO adipocytes from HFD. (C) Inguinal adipocytes from chow-fed mice were non-stimulated or stimulated with insulin (0.1 or 1 nM) for 30 min, followed by western blot analysis to detect total and phosphorylated protein levels of (A) IRS-1 (pY612), (B) AKT (pS473), (C) AS160 (pT642) and EHD2. (D) IRβ protein levels in inguinal adipocytes from chow-fed mice. Data are displayed as mean±SD, and unpaired two-sample t-test was used for statistical analysis. Significance was determined according to $^*p \leq 0.05$. n=4 biological replicates for all displayed results.

Supplemental Figure S2. Multiple Reaction Monitoring for lipidomic data.
Principal component analysis (PCA) of lipid data obtained for serum (top), fat cake (middle) and membrane fraction (below) aquired in positive (left column) and negative (right column) electrospray ionization mode (ESI^+ and ESI^-, respectively).

Supplement Figure S1

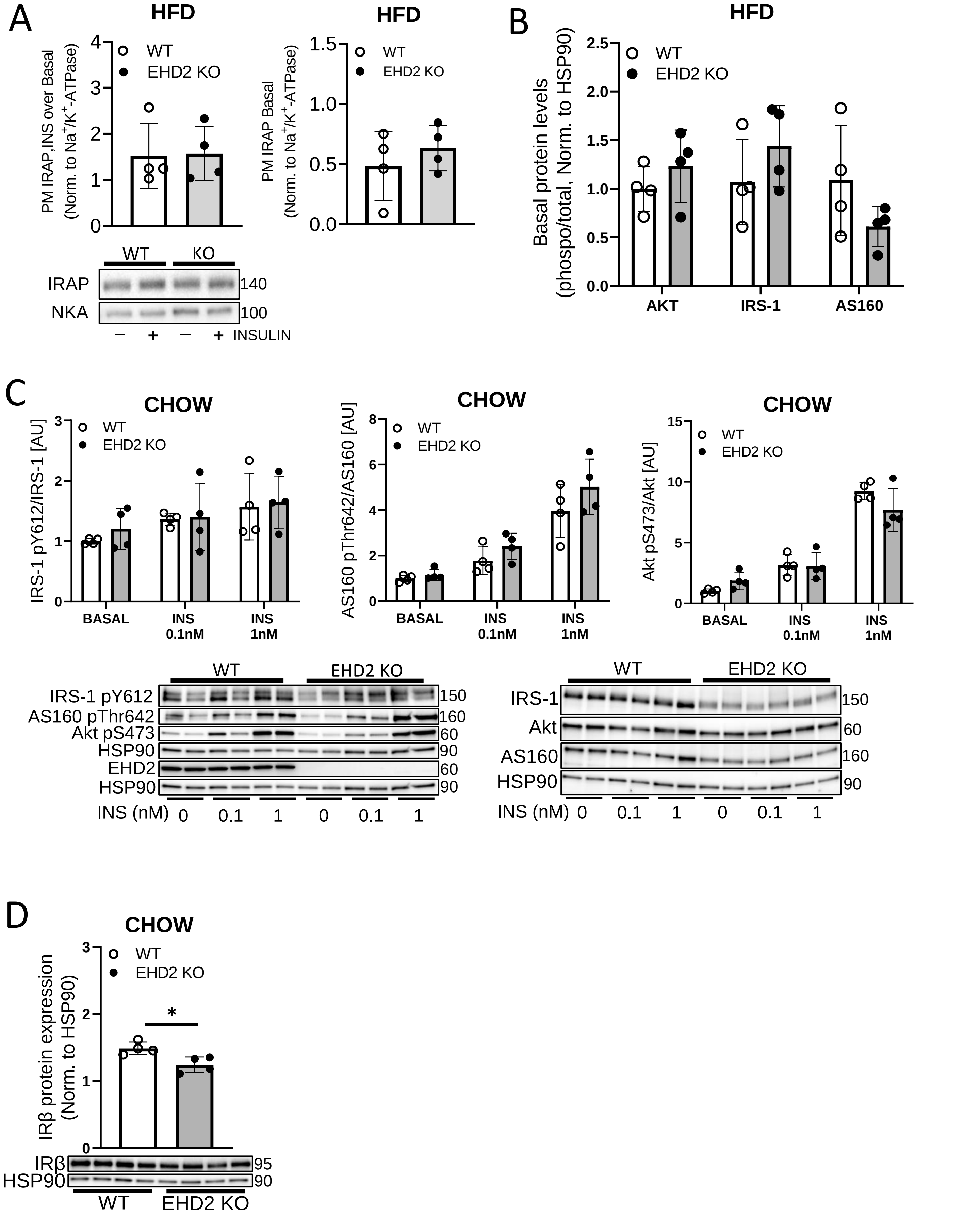

Supplement Figure S2

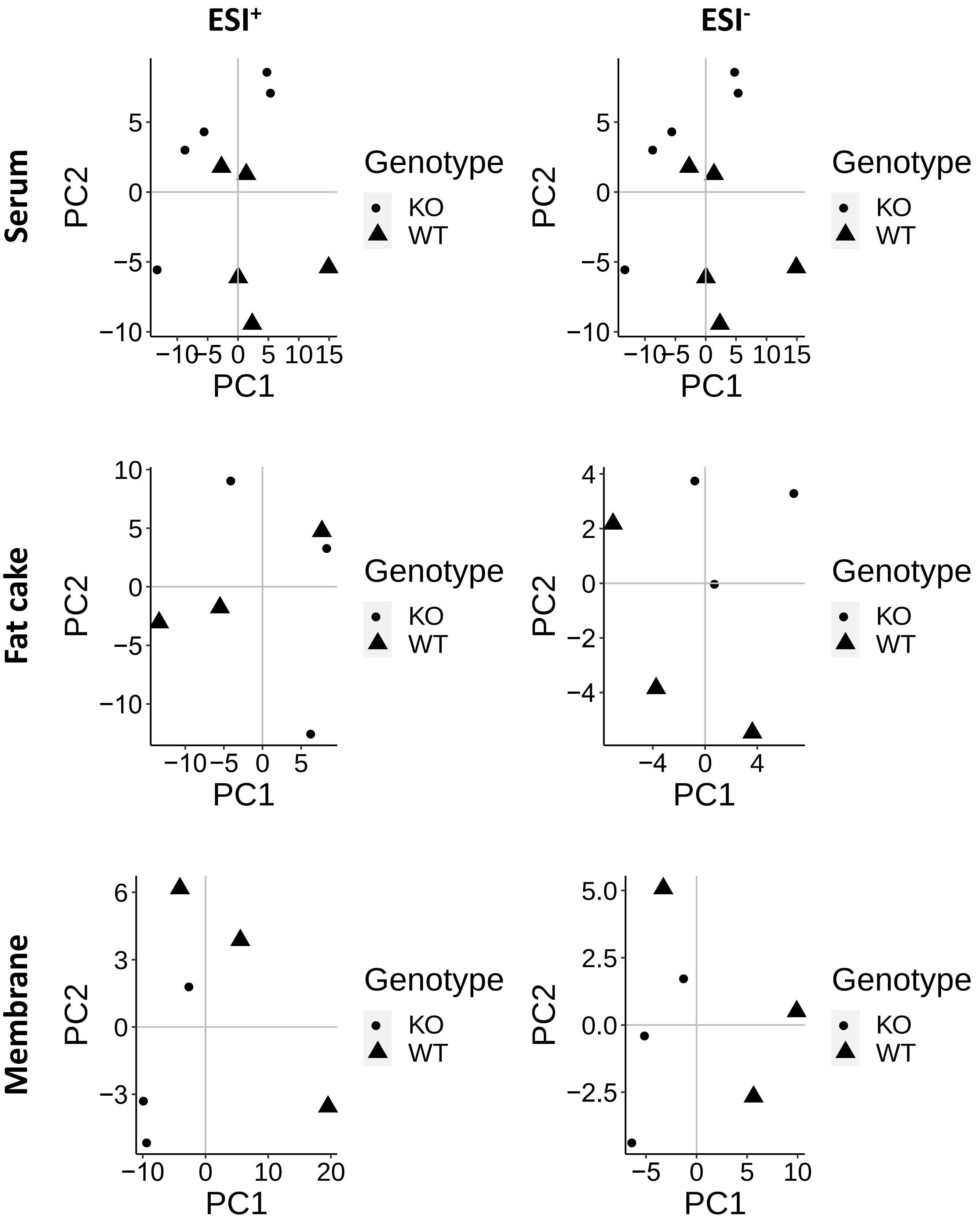

Supplemental Table S1 - Transitions applied in positive ESI mode.

N	Compound Group	Compound Name	Precursor Ion	MS1 Res	Product Ion	MS2 Res	Ret Time (min)	Delta Ret Time	Fragmentor	Collision Energy	Cell Accelerator Voltage	Polarity
1	AC	AcCar_16_0	400.40	Unit	85.03	Unit	1.5	2	380	30	5	Positive
2	AC	AcCar_18_0	428.40	Unit	85.03	Unit	1.9	2	380	30	5	Positive
3	AC	AcCar_18_1	426.40	Unit	85.03	Unit	1.5	2	380	30	5	Positive
4	AC	AcCar_18_2	424.30	Unit	85.03	Unit	1.3	2	380	30	5	Positive
5	CE	CE_16_0	642.60	Unit	369.35	Unit	9.5	2	380	10	5	Positive
6	CE	CE_16_0_d7_IS	649.66	Unit	376.40	Unit	9.2	2	380	10	5	Positive
7	CE	CE_16_1	640.60	Unit	369.35	Unit	9.4	2	380	10	5	Positive
8	CE	CE_18_0	670.64	Unit	369.35	Unit	9.9	2	380	10	5	Positive
9	CE	CE_18_1	670.64	Unit	369.35	Unit	9.9	2	380	10	5	Positive
10	CE	CE_18_2	666.62	Unit	369.35	Unit	9.3	2	380	10	5	Positive
11	CE	CE_18_3	664.61	Unit	369.35	Unit	9.2	2	380	10	5	Positive
12	CE	CE_20_1	696.67	Unit	369.35	Unit	9.9	2	380	10	5	Positive
13	CE	CE_20_2	694.65	Unit	369.35	Unit	9.7	2	380	10	5	Positive
14	CE	CE_20_3	692.63	Unit	369.35	Unit	9.5	2	380	10	5	Positive
15	CE	CE_20_4	690.62	Unit	369.35	Unit	9.3	2	380	10	5	Positive
16	CE	CE_20_5	688.60	Unit	369.35	Unit	8.6	2	380	10	5	Positive
17	CE	CE_22_1	724.70	Unit	369.35	Unit	10.1	2	380	10	5	Positive
18	CE	CE_22_2	722.68	Unit	369.35	Unit	9.9	2	380	10	5	Positive
19	CE	CE_22_3	720.67	Unit	369.35	Unit	9.7	2	380	10	5	Positive
20	CE	CE_22_4	718.65	Unit	369.35	Unit	9.6	2	380	10	5	Positive
21	CE	CE_22_5	716.63	Unit	369.35	Unit	9.4	2	380	10	5	Positive
22	CE	CE_22_6	714.62	Unit	369.35	Unit	9.1	2	380	10	5	Positive
23	Cer	Cer_d18_1_14_0	510.49	Unit	264.27	Unit	5.2	2	380	26	5	Positive
24	Cer	Cer_d18_1_16_0	538.52	Unit	264.27	Unit	5.9	2	380	26	5	Positive
25	Cer	Cer_d18_1_16_1	536.50	Unit	264.27	Unit	5.3	2	380	26	5	Positive
26	Cer	Cer_d18_1_18_0	566.55	Unit	264.27	Unit	6.5	2	380	26	5	Positive
27	Cer	Cer_d18_1_20_0	594.58	Unit	264.27	Unit	7.0	2	380	26	5	Positive
28	Cer	Cer_d18_1_20_1	592.57	Unit	264.27	Unit	6.2	2	380	26	5	Positive
29	Cer	Cer_d18_1_22_0	622.61	Unit	264.27	Unit	7.5	2	380	26	5	Positive
30	Cer	Cer_d18_1_22_1	620.60	Unit	264.27	Unit	7.0	2	380	26	5	Positive
31	Cer	Cer_d18_1_23_0	636.63	Unit	264.27	Unit	7.7	2	380	26	5	Positive
32	Cer	Cer_d18_1_23_1	634.61	Unit	264.27	Unit	7.2	2	380	26	5	Positive
33	Cer	Cer_d18_1_24_0	650.64	Unit	264.27	Unit	7.9	2	380	26	5	Positive
34	Cer	Cer_d18_1_24_1	648.63	Unit	264.27	Unit	7.4	2	380	26	5	Positive
35	Cer	Cer_d18_1_26_0	678.68	Unit	264.27	Unit	8.3	2	380	26	5	Positive
36	Cer	Cer_d18_1_28_0	706.71	Unit	264.27	Unit	8.8	2	380	26	5	Positive
37	DAG	DAG_14_0_14_0_IS	530.50	Unit	285.24	Unit	5.7	2	380	22	5	Positive
38	DAG	DAG_14_0_16_0	558.50	Unit	285.24	Unit	6.3	2	380	22	5	Positive
39	DAG	DAG_14_0_16_1	556.50	Unit	285.24	Unit	5.7	2	380	22	5	Positive
40	DAG	DAG_14_0_18_1	584.50	Unit	285.24	Unit	6.3	2	380	22	5	Positive
41	DAG	DAG_16_0_16_0	586.50	Unit	313.27	Unit	6.8	2	380	22	5	Positive
42	DAG	DAG_16_0_16_1	584.50	Unit	313.27	Unit	6.4	2	380	22	5	Positive
43	DAG	DAG_16_0_18_0	614.60	Unit	313.27	Unit	7.3	2	380	22	5	Positive
44	DAG	DAG_16_0_18_1	612.60	Unit	313.27	Unit	6.9	2	380	22	5	Positive
45	DAG	DAG_16_0_18_2	610.50	Unit	313.27	Unit	6.4	2	380	22	5	Positive
46	DAG	DAG_16_0_18_3	608.50	Unit	313.27	Unit	6.2	2	380	22	5	Positive
47	DAG	DAG_16_0_20_2	638.60	Unit	313.27	Unit	6.9	2	380	22	5	Positive
48	DAG	DAG_16_0_20_3	636.60	Unit	313.27	Unit	6.7	2	380	22	5	Positive
49	DAG	DAG_16_0_20_4	634.50	Unit	313.27	Unit	6.3	2	380	22	5	Positive
50	DAG	DAG_16_1_16_1	582.50	Unit	311.26	Unit	5.9	2	380	22	5	Positive
51	DAG	DAG_16_1_18_0	612.60	Unit	311.26	Unit	6.9	2	380	22	5	Positive
52	DAG	DAG_16_1_18_1	610.50	Unit	311.26	Unit	6.4	2	380	22	5	Positive
53	DAG	DAG_16_1_18_2	608.50	Unit	311.26	Unit	6.0	2	380	22	5	Positive

54	DAG	DAG_16_1_20_2	636.60	Unit	311.26	Unit	6.5	2	380	22	5	Positive
55	DAG	DAG_16_1_20_3	634.50	Unit	311.26	Unit	6.2	2	380	22	5	Positive
56	DAG	DAG_18_0_18_0	642.60	Unit	341.31	Unit	7.7	2	380	22	5	Positive
57	DAG	DAG_18_0_18_1	640.60	Unit	341.31	Unit	7.4	2	380	22	5	Positive
58	DAG	DAG_18_0_18_2	638.60	Unit	341.31	Unit	7.0	2	380	22	5	Positive
59	DAG	DAG_18_0_18_3	636.60	Unit	341.31	Unit	6.7	2	380	22	5	Positive
60	DAG	DAG_18_0_20_4	662.60	Unit	341.31	Unit	6.8	2	380	22	5	Positive
61	DAG	DAG_18_1_18_1	638.60	Unit	339.29	Unit	6.9	2	380	22	5	Positive
62	DAG	DAG_18_1_18_2	636.60	Unit	339.29	Unit	6.5	2	380	22	5	Positive
63	DAG	DAG_18_1_18_3	634.50	Unit	339.29	Unit	6.2	2	380	22	5	Positive
64	DAG	DAG_18_1_20_1	666.60	Unit	339.29	Unit	7.3	2	380	22	5	Positive
65	DAG	DAG_18_1_20_2	664.60	Unit	339.29	Unit	7.0	2	380	22	5	Positive
66	DAG	DAG_18_1_20_4	660.60	Unit	339.29	Unit	6.3	2	380	22	5	Positive
67	LPC	LPC_12_0	440.28	Unit	184.07	Unit	1.0	2	380	22	5	Positive
68	LPC	LPC_14_0	468.31	Unit	184.07	Unit	1.2	2	380	22	5	Positive
69	LPC	LPC_16_0	496.34	Unit	184.07	Unit	1.7	2	380	22	5	Positive
70	LPC	LPC_16_1	494.32	Unit	184.07	Unit	1.3	2	380	22	5	Positive
71	LPC	LPC_17_0	510.36	Unit	184.07	Unit	1.9	2	380	22	5	Positive
72	LPC	LPC_18_0	524.37	Unit	184.07	Unit	2.3	2	380	22	5	Positive
73	LPC	LPC_18_1	522.36	Unit	184.07	Unit	1.7	2	380	22	5	Positive
74	LPC	LPC_18_2	520.34	Unit	184.07	Unit	1.4	2	380	22	5	Positive
75	LPC	LPC_18_3	518.32	Unit	184.07	Unit	1.2	2	380	22	5	Positive
76	LPC	LPC_20_1	550.39	Unit	184.07	Unit	2.3	2	380	22	5	Positive
77	LPC	LPC_20_2	548.37	Unit	184.07	Unit	1.8	2	380	22	5	Positive
78	LPC	LPC_20_3	546.36	Unit	184.07	Unit	1.6	2	380	22	5	Positive
79	LPC	LPC_20_4	544.34	Unit	184.07	Unit	1.3	2	380	22	5	Positive
80	LPC	LPC_20_5	542.32	Unit	184.07	Unit	1.1	2	380	22	5	Positive
81	LPC	LPC_22_1	578.42	Unit	184.07	Unit	2.9	2	380	22	5	Positive
82	LPC	LPC_22_2	576.40	Unit	184.07	Unit	2.3	2	380	22	5	Positive
83	LPC	LPC_22_3	574.39	Unit	184.07	Unit	2.0	2	380	22	5	Positive
84	LPC	LPC_22_4	572.37	Unit	184.07	Unit	1.8	2	380	22	5	Positive
85	LPC	LPC_22_5	570.36	Unit	184.07	Unit	1.5	2	380	22	5	Positive
86	LPC	LPC_22_6	568.34	Unit	184.07	Unit	1.3	2	380	22	5	Positive
87	PC_e_p	PC_16_0_e_p_20_4	768.60	Unit	482.36	Unit	6.0	2	380	30	5	Positive
88	PC_e_p	PC_16_1_e_p_20_4	766.60	Unit	480.34	Unit	5.4	2	380	30	5	Positive
89	PC_e_p	PC_16_1_e_p_22_6	790.57	Unit	480.34	Unit	5.3	2	380	30	5	Positive
90	PC_e_p	PC_16_2_e_p_22_6	788.60	Unit	550.33	Unit	6.3	2	380	30	5	Positive
91	PC	PC_24_0_IS	622.44	Unit	184.07	Unit	3.0	2	380	30	5	Positive
92	PC	PC_28_0	678.51	Unit	184.07	Unit	4.4	2	380	30	5	Positive
93	PC	PC_32_0	734.57	Unit	184.07	Unit	5.8	2	380	30	5	Positive
94	PC	PC_32_1	732.55	Unit	184.07	Unit	5.2	2	380	30	5	Positive
95	PC	PC_34_0	762.60	Unit	184.07	Unit	6.3	2	380	30	5	Positive
96	PC	PC_34_1	760.59	Unit	184.07	Unit	5.8	2	380	30	5	Positive
97	PC	PC_34_2	758.57	Unit	184.07	Unit	5.4	2	380	30	5	Positive
98	PC	PC_34_3	756.55	Unit	184.07	Unit	5.0	2	380	30	5	Positive
99	PC	PC_36_0	790.63	Unit	184.07	Unit	7.0	2	380	30	5	Positive
100	PC	PC_36_1	788.62	Unit	184.07	Unit	6.4	2	380	30	5	Positive
101	PC	PC_36_2	786.60	Unit	184.07	Unit	5.9	2	380	30	5	Positive
102	PC	PC_36_3	784.59	Unit	184.07	Unit	5.7	2	380	30	5	Positive
103	PC	PC_36_4	782.57	Unit	184.07	Unit	5.2	2	380	30	5	Positive
104	PC	PC_36_5	780.55	Unit	184.07	Unit	4.7	2	380	30	5	Positive
105	PC	PC_38_2	814.63	Unit	184.07	Unit	6.9	2	380	30	5	Positive
106	PC	PC_38_3	812.61	Unit	184.07	Unit	6.2	2	380	30	5	Positive
107	PC	PC_38_4	810.60	Unit	184.07	Unit	5.7	2	380	30	5	Positive
108	PC	PC_38_5	808.59	Unit	184.07	Unit	5.2	2	380	30	5	Positive
109	PC	PC_38_6	806.57	Unit	184.07	Unit	5.0	2	380	30	5	Positive
110	PC	PC_40_2	842.66	Unit	184.07	Unit	6.9	2	380	30	5	Positive
111	PC	PC_40_3	840.65	Unit	184.07	Unit	6.4	2	380	30	5	Positive

112	PC	PC_40_4	838.63	Unit	184.07	Unit	6.1	2	380	30	5	Positive
113	PC	PC_40_5	836.62	Unit	184.07	Unit	5.8	2	380	30	5	Positive
114	PC	PC_40_6	834.60	Unit	184.07	Unit	5.6	2	380	30	5	Positive
115	PE p	PE_16_0_p_16_1	674.50	Unit	311.26	Unit	6.0	2	380	30	5	Positive
116	PE p	PE_16_0_p_18_1	702.50	Unit	339.29	Unit	6.3	2	380	30	5	Positive
117	PE p	PE_16_0_p_20_3	726.50	Unit	363.29	Unit	6.1	2	380	30	5	Positive
118	PE p	PE_16_0_p_20_4	724.50	Unit	361.27	Unit	5.6	2	380	30	5	Positive
119	PE p	PE_16_0_p_22_5	750.50	Unit	387.29	Unit	5.6	2	380	30	5	Positive
120	PE p	PE_16_0_p_22_6	748.50	Unit	385.27	Unit	5.5	2	380	30	5	Positive
121	PE p	PE_18_0_p_16_1	702.50	Unit	311.26	Unit	6.3	2	380	30	5	Positive
122	PE p	PE_18_0_p_18_1	730.60	Unit	339.29	Unit	6.8	2	380	30	5	Positive
123	PE p	PE_18_0_p_20_3	754.60	Unit	363.29	Unit	6.6	2	380	30	5	Positive
124	PE p	PE_18_0_p_20_4	752.60	Unit	361.27	Unit	6.2	2	380	30	5	Positive
125	PE p	PE_18_0_p_22_5	778.60	Unit	387.29	Unit	6.4	2	380	30	5	Positive
126	PE p	PE_18_0_p_22_6	776.60	Unit	385.27	Unit	6.1	2	380	30	5	Positive
127	PE p	PE_18_1_p_16_1	700.50	Unit	311.26	Unit	5.7	2	380	30	5	Positive
128	PE p	PE_18_1_p_18_1	728.60	Unit	339.29	Unit	6.3	2	380	30	5	Positive
129	PE p	PE_18_1_p_20_3	752.60	Unit	363.29	Unit	6.1	2	380	30	5	Positive
130	PE p	PE_18_1_p_20_4	750.50	Unit	361.27	Unit	5.7	2	380	30	5	Positive
131	PE p	PE_18_1_p_20_5	748.50	Unit	359.26	Unit	5.2	2	380	30	5	Positive
132	PE p	PE_18_1_p_22_5	776.60	Unit	387.29	Unit	5.7	2	380	30	5	Positive
133	PE p	PE_18_1_p_22_6	774.50	Unit	385.27	Unit	5.5	2	380	30	5	Positive
134	SM	SM_d18_0_18_0	733.62	Unit	184.07	Unit	5.2	2	380	30	5	Positive
135	SM	SM_d18_0_20_0	761.65	Unit	184.07	Unit	5.8	2	380	30	5	Positive
136	SM	SM_d18_0_22_0	789.68	Unit	184.07	Unit	6.4	2	380	30	5	Positive
137	SM	SM_d18_1_14_0	675.54	Unit	184.07	Unit	4.4	2	380	30	5	Positive
138	SM	SM_d18_1_14_1	673.53	Unit	184.07	Unit	3.8	2	380	30	5	Positive
139	SM	SM_d18_1_15_0	689.56	Unit	184.07	Unit	4.8	2	380	30	5	Positive
140	SM	SM_d18_1_16_0	703.57	Unit	184.07	Unit	5.1	2	380	30	5	Positive
141	SM	SM_d18_1_16_1	701.56	Unit	184.07	Unit	4.5	2	380	30	5	Positive
142	SM	SM_d18_1_17_0	717.59	Unit	184.07	Unit	5.5	2	380	30	5	Positive
143	SM	SM_d18_1_18_0	731.61	Unit	184.07	Unit	5.8	2	380	30	5	Positive
144	SM	SM_d18_1_18_1	729.59	Unit	184.07	Unit	5.2	2	380	30	5	Positive
145	SM	SM_d18_1_20_0	759.64	Unit	184.07	Unit	5.3	2	380	30	5	Positive
146	SM	SM_d18_1_20_1	757.62	Unit	184.07	Unit	5.1	2	380	30	5	Positive
147	SM	SM_d18_1_20_2	755.61	Unit	184.07	Unit	4.5	2	380	30	5	Positive
148	SM	SM_d18_1_22_0	787.67	Unit	184.07	Unit	5.8	2	380	30	5	Positive
149	SM	SM_d18_1_22_1	785.65	Unit	184.07	Unit	5.7	2	380	30	5	Positive
150	SM	SM_d18_1_22_2	783.64	Unit	184.07	Unit	5.2	2	380	30	5	Positive
151	SM	SM_d18_1_22_3	781.62	Unit	184.07	Unit	4.7	2	380	30	5	Positive
152	SM	SM_d18_1_23_0	801.68	Unit	184.07	Unit	7.2	2	380	30	5	Positive
153	SM	SM_d18_1_23_1	799.67	Unit	184.07	Unit	6.6	2	380	30	5	Positive
154	SM	SM_d18_1_24_0	815.70	Unit	184.07	Unit	7.4	2	380	30	5	Positive
155	SM	SM_d18_1_24_1	813.68	Unit	184.07	Unit	6.9	2	380	30	5	Positive
156	SM	SM_d18_1_24_2	811.67	Unit	184.07	Unit	6.4	2	380	30	5	Positive
157	SM	SM_d18_1_25_0	829.72	Unit	184.07	Unit	7.5	2	380	30	5	Positive
158	SM	SM_d18_1_25_1	827.70	Unit	184.07	Unit	7.1	2	380	30	5	Positive
159	SM	SM_d18_1_26_1	841.72	Unit	184.07	Unit	7.3	2	380	30	5	Positive
160	TG	TG_36_0_IS	656.60	Unit	656.60	Unit	7.4	2	380	10	5	Positive
161	TG	TG_42_0	740.70	Unit	740.70	Unit	8.5	2	380	10	5	Positive
162	TG	TG_44_0	768.70	Unit	768.70	Unit	8.8	2	380	10	5	Positive
163	TG	TG_44_1	766.70	Unit	766.70	Unit	8.5	2	380	10	5	Positive
164	TG	TG_46_0	796.70	Unit	796.70	Unit	9.1	2	380	10	5	Positive
165	TG	TG_46_1	794.70	Unit	794.70	Unit	8.8	2	380	10	5	Positive
166	TG	TG_46_2	792.70	Unit	792.70	Unit	8.6	2	380	10	5	Positive
167	TG	TG_48_0	824.80	Unit	824.80	Unit	9.4	2	380	10	5	Positive
168	TG	TG_48_1	822.80	Unit	822.80	Unit	9.1	2	380	10	5	Positive
169	TG	TG_48_2	820.70	Unit	820.70	Unit	8.9	2	380	10	5	Positive

170	TG	TG_48_3	818.70	Unit	818.70	Unit	8.6	2	380	10	5	Positive
171	TG	TG_50_0	852.80	Unit	852.80	Unit	9.6	2	380	10	5	Positive
172	TG	TG_50_1	850.80	Unit	850.80	Unit	9.4	2	380	10	5	Positive
173	TG	TG_50_2	848.80	Unit	848.80	Unit	9.1	2	380	10	5	Positive
174	TG	TG_50_3	846.80	Unit	846.80	Unit	8.9	2	380	10	5	Positive
175	TG	TG_50_4	844.70	Unit	844.70	Unit	8.7	2	380	10	5	Positive
176	TG	TG_52_1	878.80	Unit	878.80	Unit	9.6	2	380	10	5	Positive
177	TG	TG_52_2	876.80	Unit	876.80	Unit	9.4	2	380	10	5	Positive
178	TG	TG_52_3	874.80	Unit	874.80	Unit	9.2	2	380	10	5	Positive
179	TG	TG_52_4	872.80	Unit	872.80	Unit	8.9	2	380	10	5	Positive
180	TG	TG_52_5	870.80	Unit	870.80	Unit	8.8	2	380	10	5	Positive
181	TG	TG_54_0	908.90	Unit	908.90	Unit	10.0	2	380	10	5	Positive
182	TG	TG_54_1	906.90	Unit	906.90	Unit	9.8	2	380	10	5	Positive
183	TG	TG_54_2	904.80	Unit	904.80	Unit	9.6	2	380	10	5	Positive
184	TG	TG_54_3	902.80	Unit	902.80	Unit	9.4	2	380	10	5	Positive
185	TG	TG_54_4	900.80	Unit	900.80	Unit	9.2	2	380	10	5	Positive
186	TG	TG_54_5	898.80	Unit	898.80	Unit	9.1	2	380	10	5	Positive
187	TG	TG_56_1	934.90	Unit	934.90	Unit	10.0	2	380	10	5	Positive
188	TG	TG_56_2	932.90	Unit	932.90	Unit	10.1	2	380	10	5	Positive
189	TG	TG_56_3	930.90	Unit	930.90	Unit	9.8	2	380	10	5	Positive
190	TG	TG_56_4	928.80	Unit	928.80	Unit	9.6	2	380	10	5	Positive
191	TG	TG_56_5	926.80	Unit	926.80	Unit	9.3	2	380	10	5	Positive
192	TG	TG_56_6	924.80	Unit	924.80	Unit	9.1	2	380	10	5	Positive
193	TG	TG_56_7	922.80	Unit	922.80	Unit	8.9	2	380	10	5	Positive
194	TG	TG_58_2	960.90	Unit	960.90	Unit	10.0	2	380	10	5	Positive
195	TG	TG_58_3	958.90	Unit	958.90	Unit	10.1	2	380	10	5	Positive
196	TG	TG_58_4	956.90	Unit	956.90	Unit	9.8	2	380	10	5	Positive
197	TG	TG_58_5	954.90	Unit	954.90	Unit	9.5	2	380	10	5	Positive
198	TG	TG_58_6	952.80	Unit	952.80	Unit	9.3	2	380	10	5	Positive
199	TG	TG_58_7	950.80	Unit	950.80	Unit	9.1	2	380	10	5	Positive

Manuscript Figure

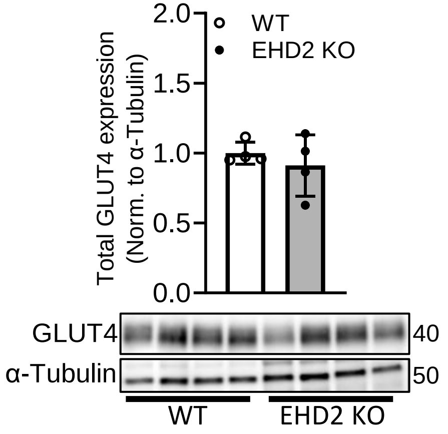

FIGURE 1E GLUT4 Total level

Total GLUT4 levels; Whole membrane for n=1-4 biological replicates(Basal only)

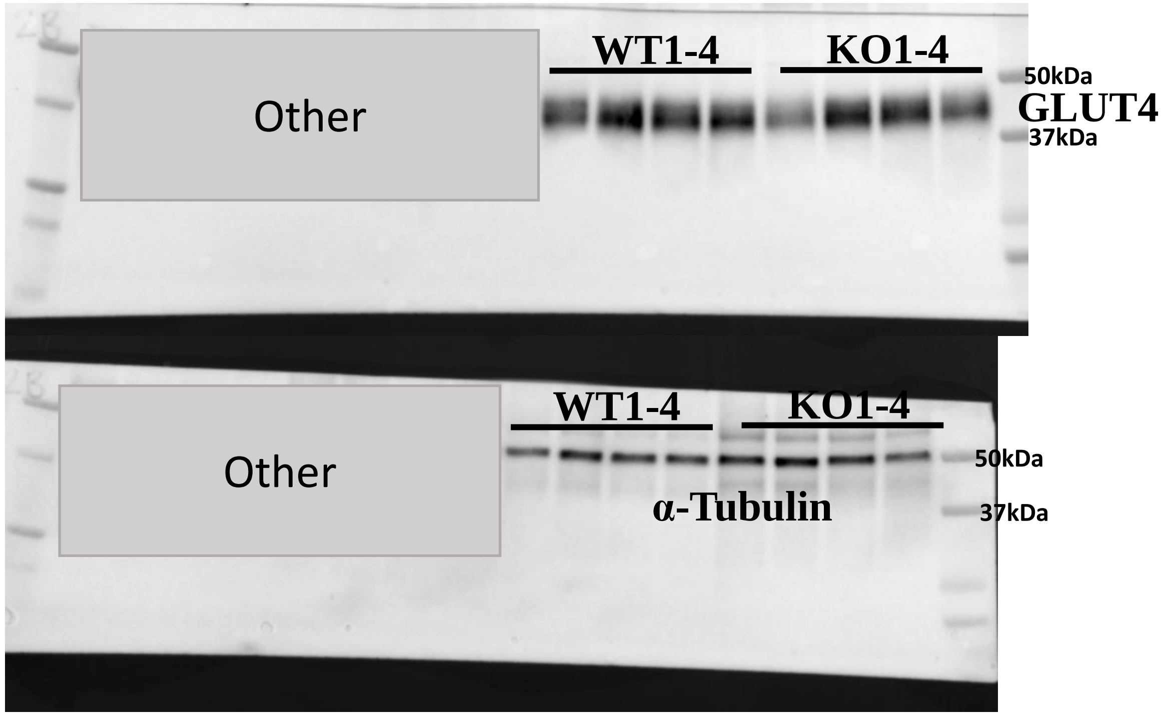

Manuscript Figure

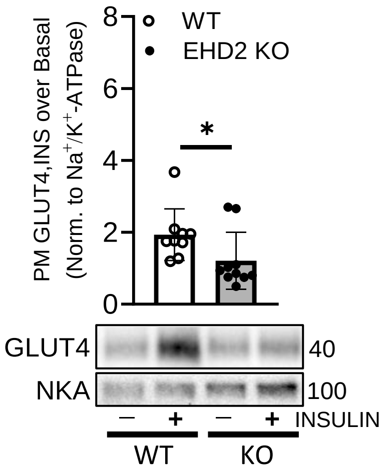

FIGURE 1F GLUT4 Translocation

+= 1nM Insulin 30 min; -= Basal; WT= wildtype, KO= EHD2 KO

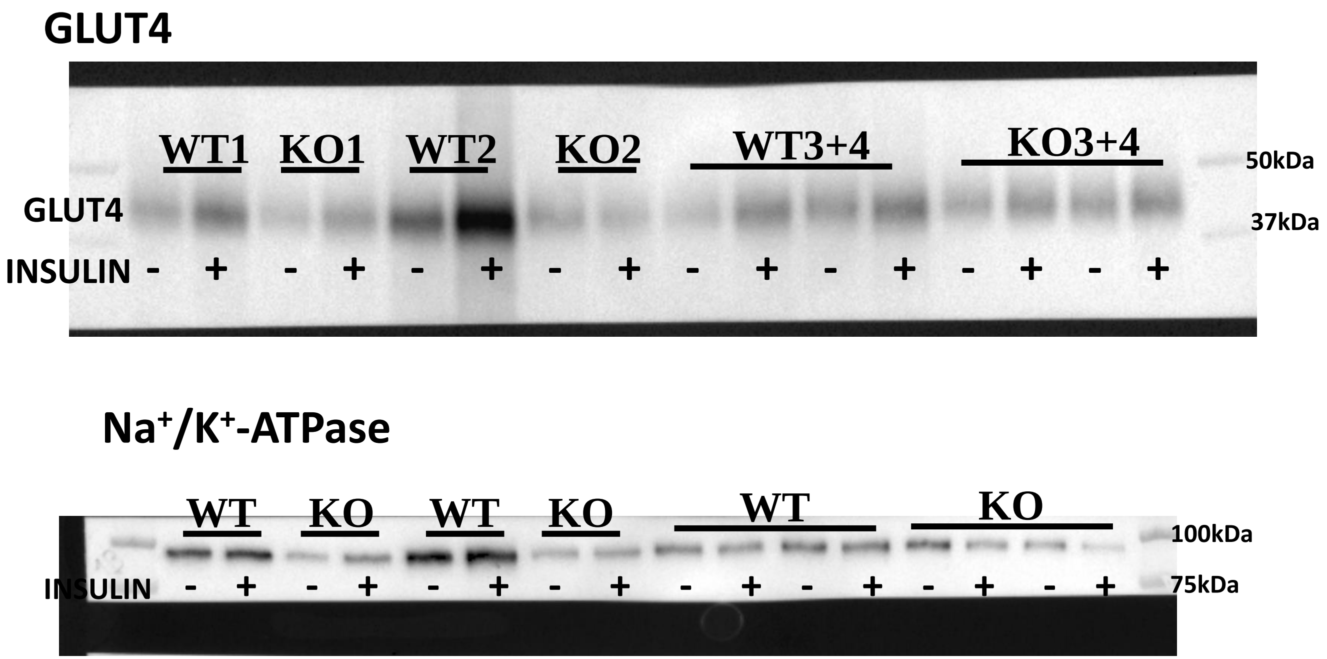

1-2 refers to biological replicates

GLUT4

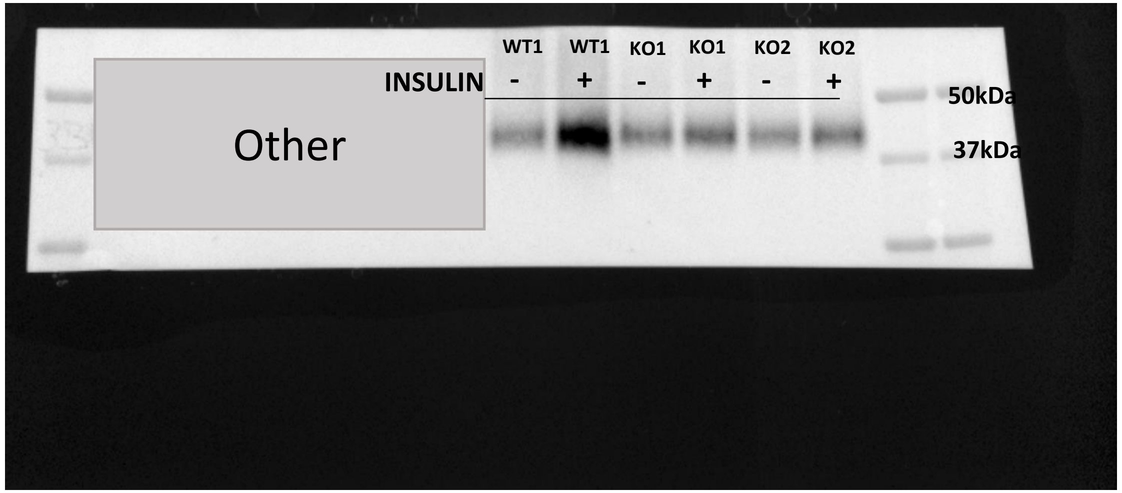

Na^+/K^+-ATPase

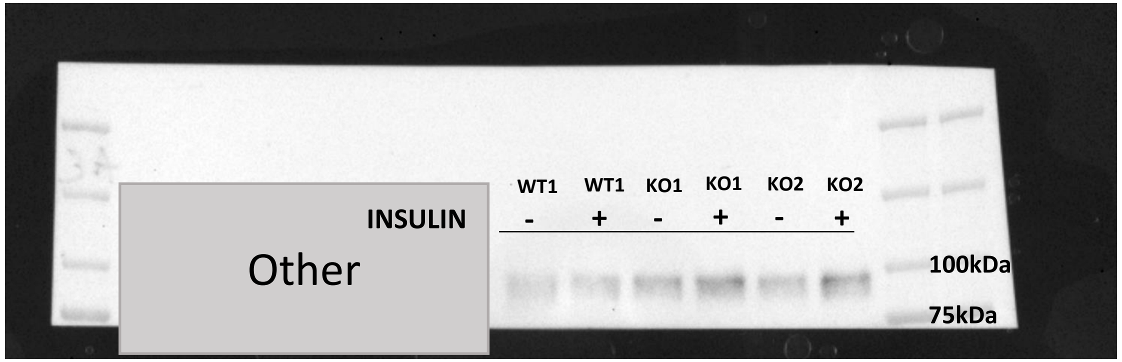

GLUT4

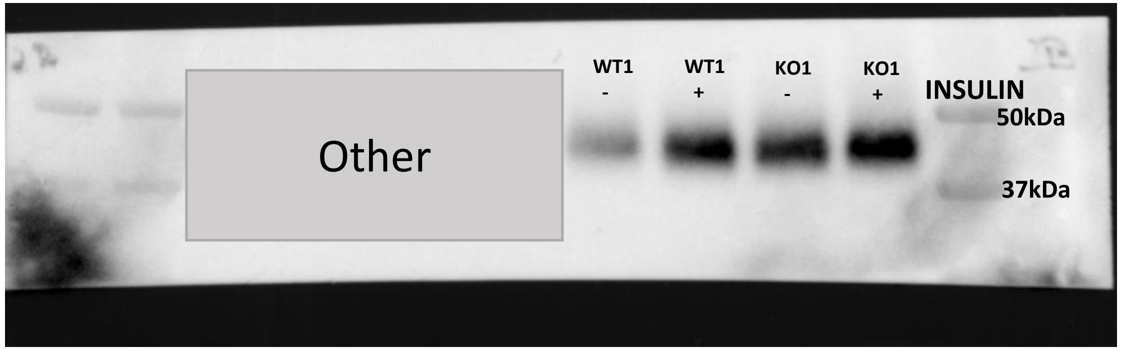

Na$^+$/K$^+$-ATPase

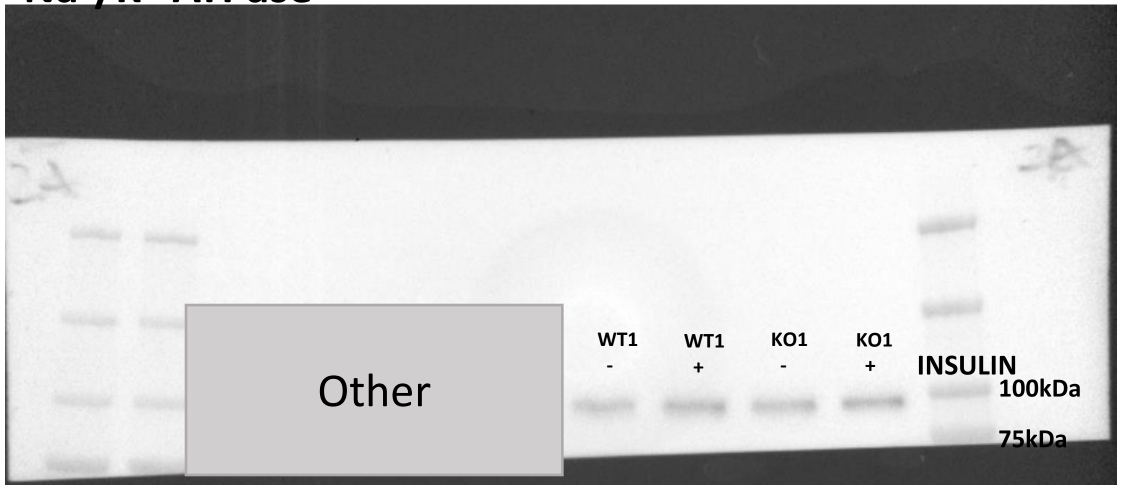

GLUT-4

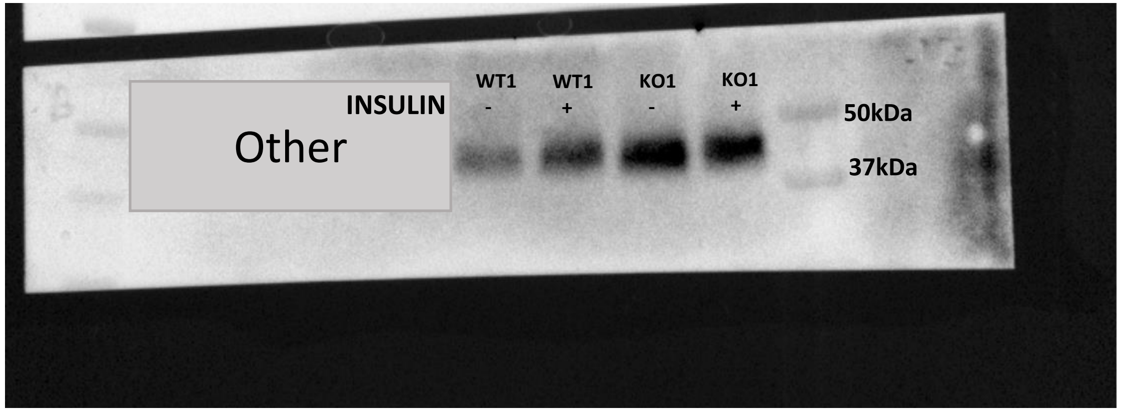

Na^+/K^+-ATPase

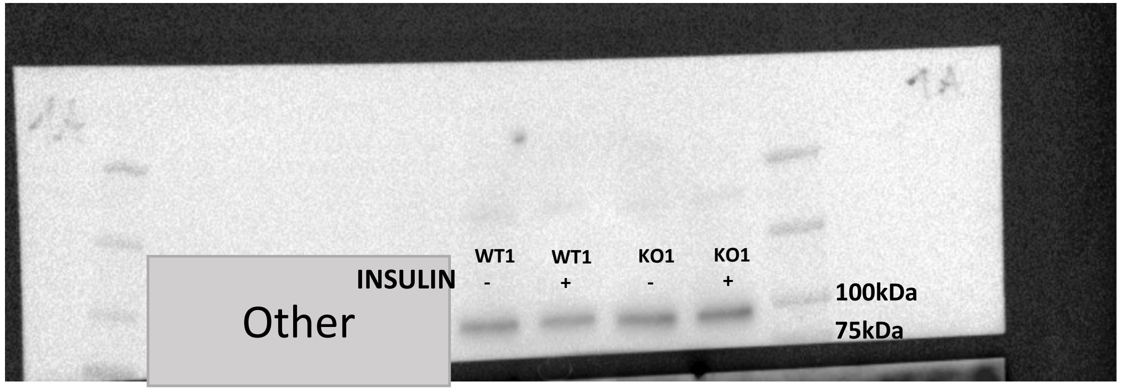

GLUT4

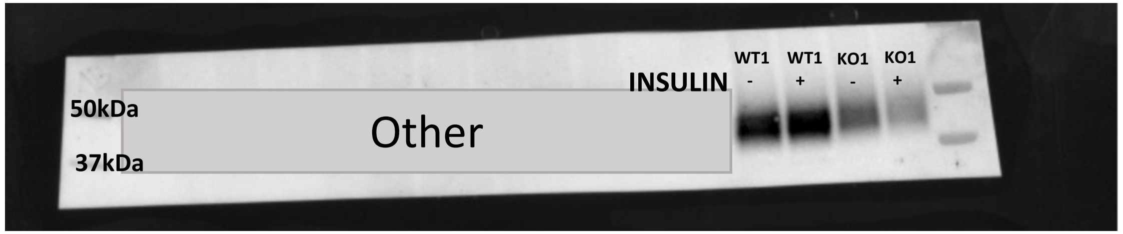

Na^+/K^+-ATPase

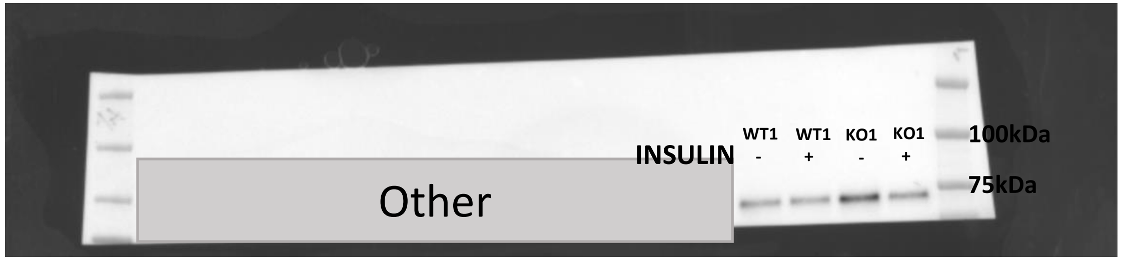

GLUT4

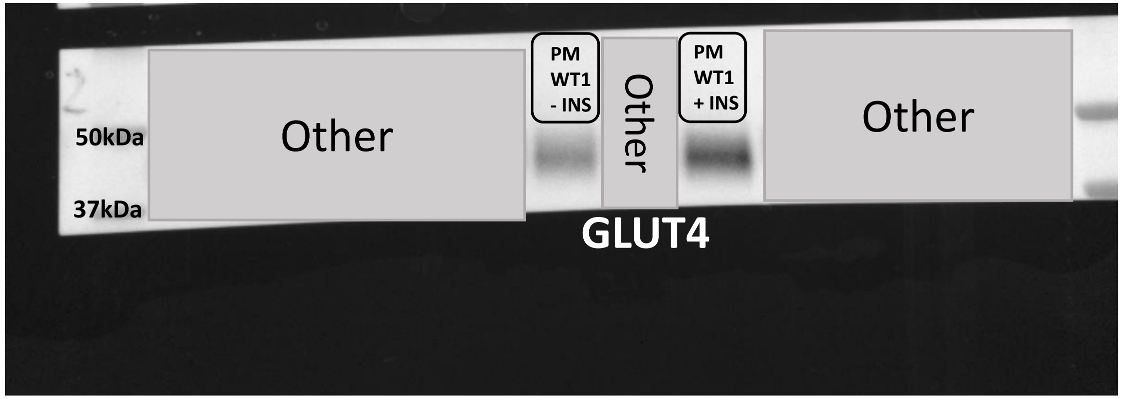

Na$^+$/K$^+$-ATPase

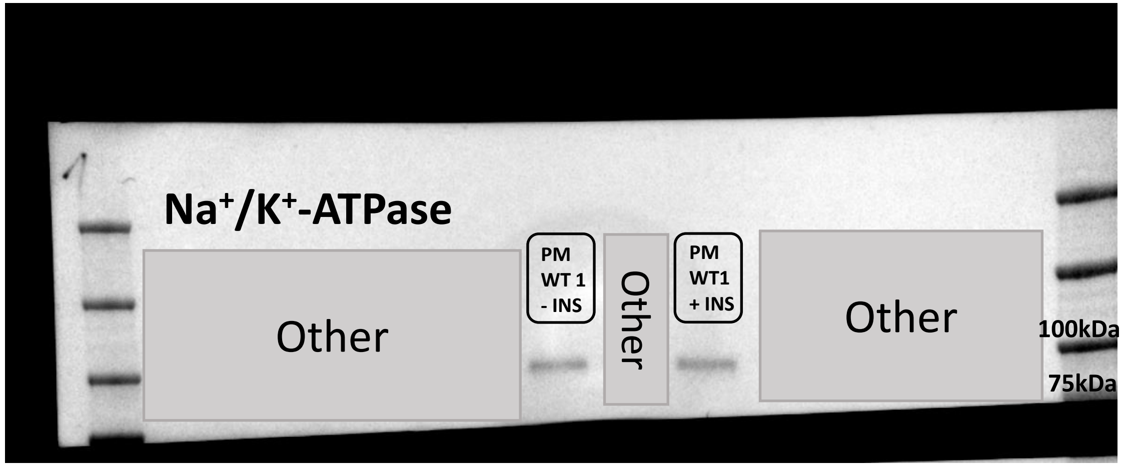

*PM= plasma membrane

GLUT4

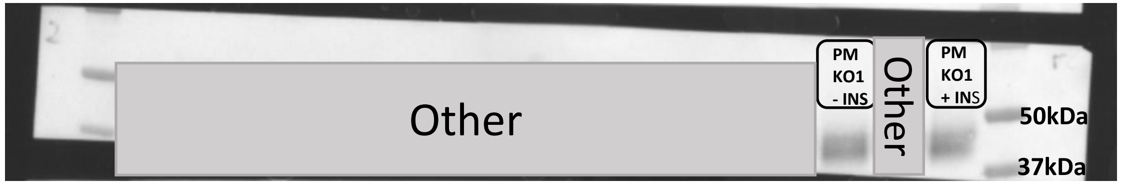

Na$^+$/K$^+$-ATPase

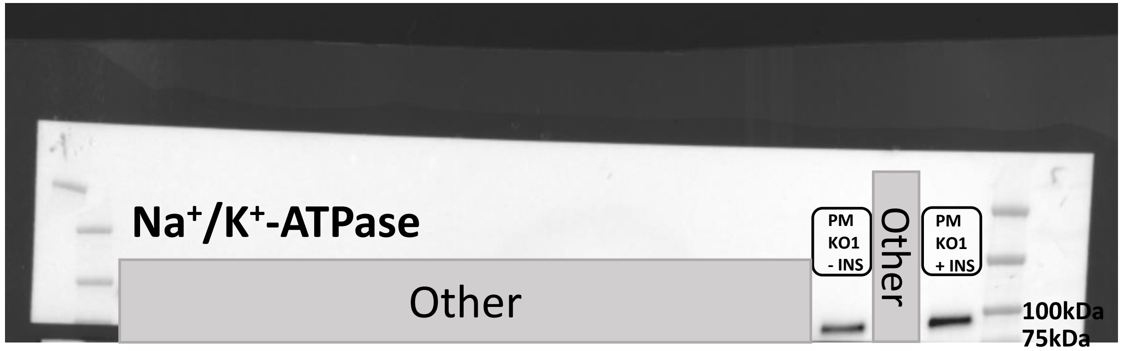

*PM= plasma membrane

Manuscript Figure

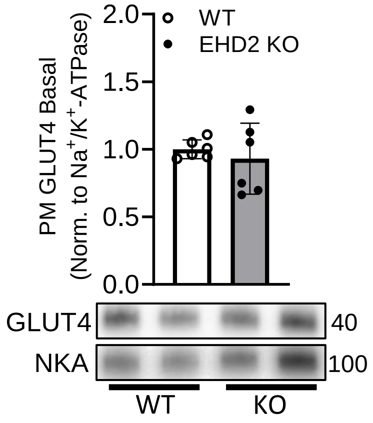

FIGURE 1G GLUT4 Plasma membrane level

Plasma membrane GLUT4 levels; Whole membrane n=6 biological replicates (Basal only)

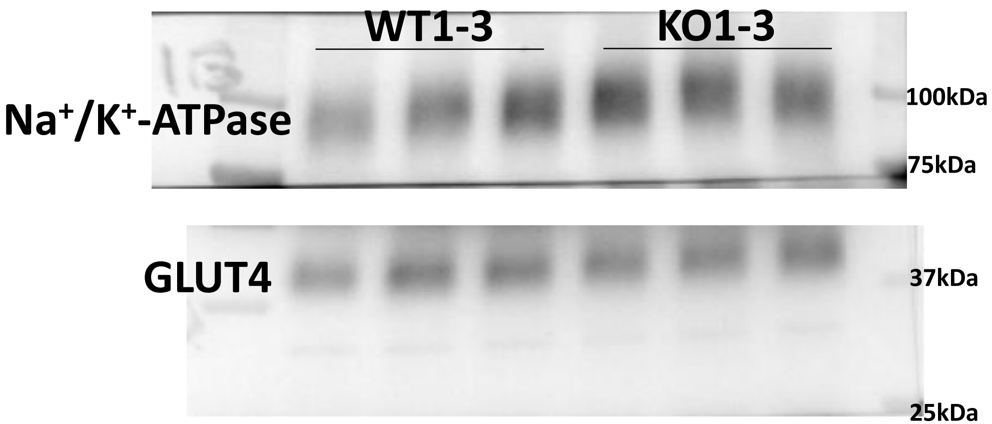

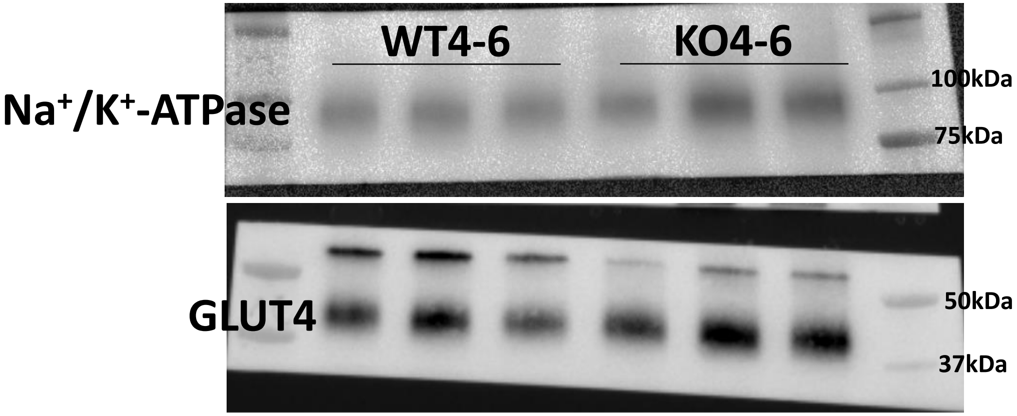

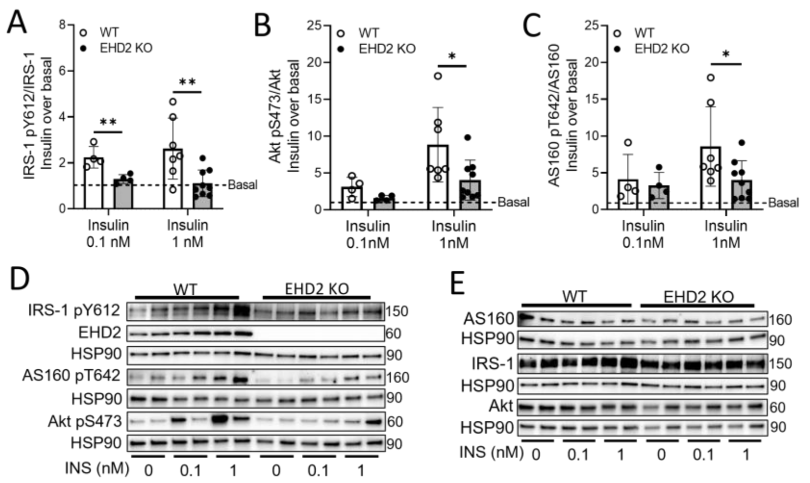

Figure 2 Insulin signaling

Whole membrane 1 and 2 for n=2 (Basal, 0.1nM and 1nM)

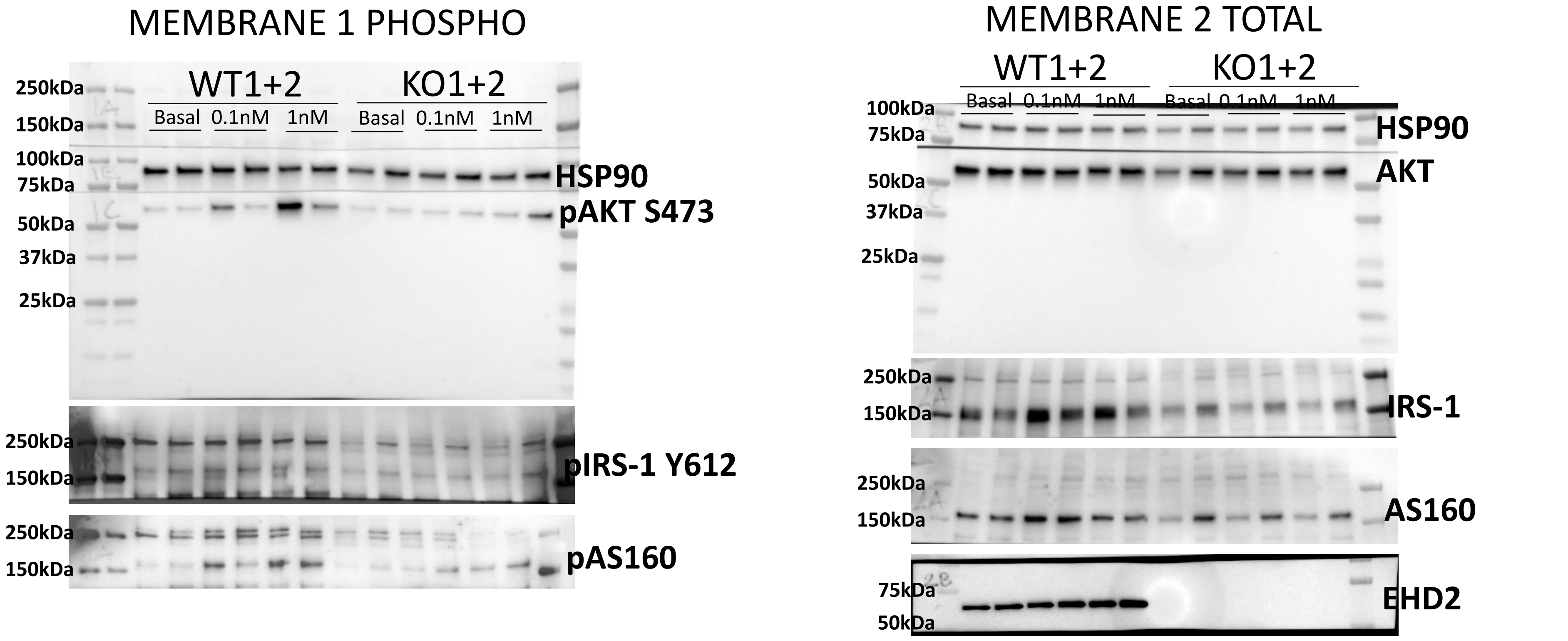

Whole membrane 3 and 4 for n=2 (Basal, 0.1nM and 1nM)

MEMBRANE 3 PHOSPHO

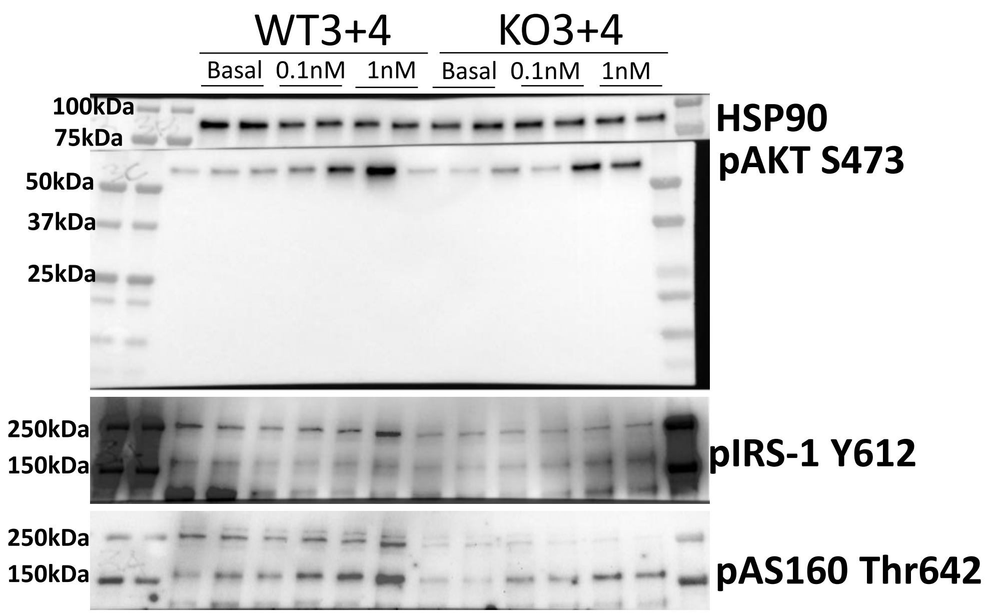

MEMBRANE 4 TOTAL

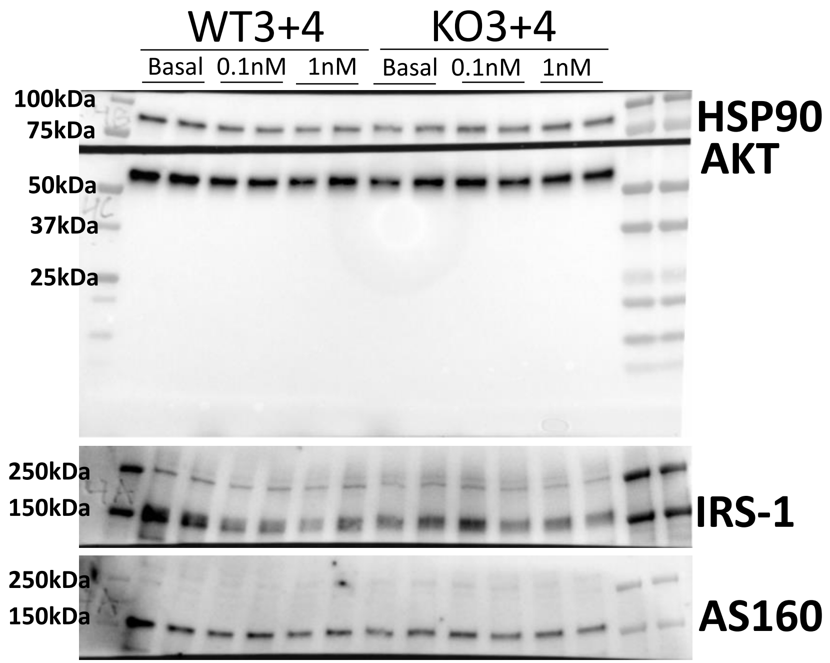

PHOSPHO, whole membranes for n=3 (WT) and n=5 (KO) biological replicates (ONLY Basal and 1nM)

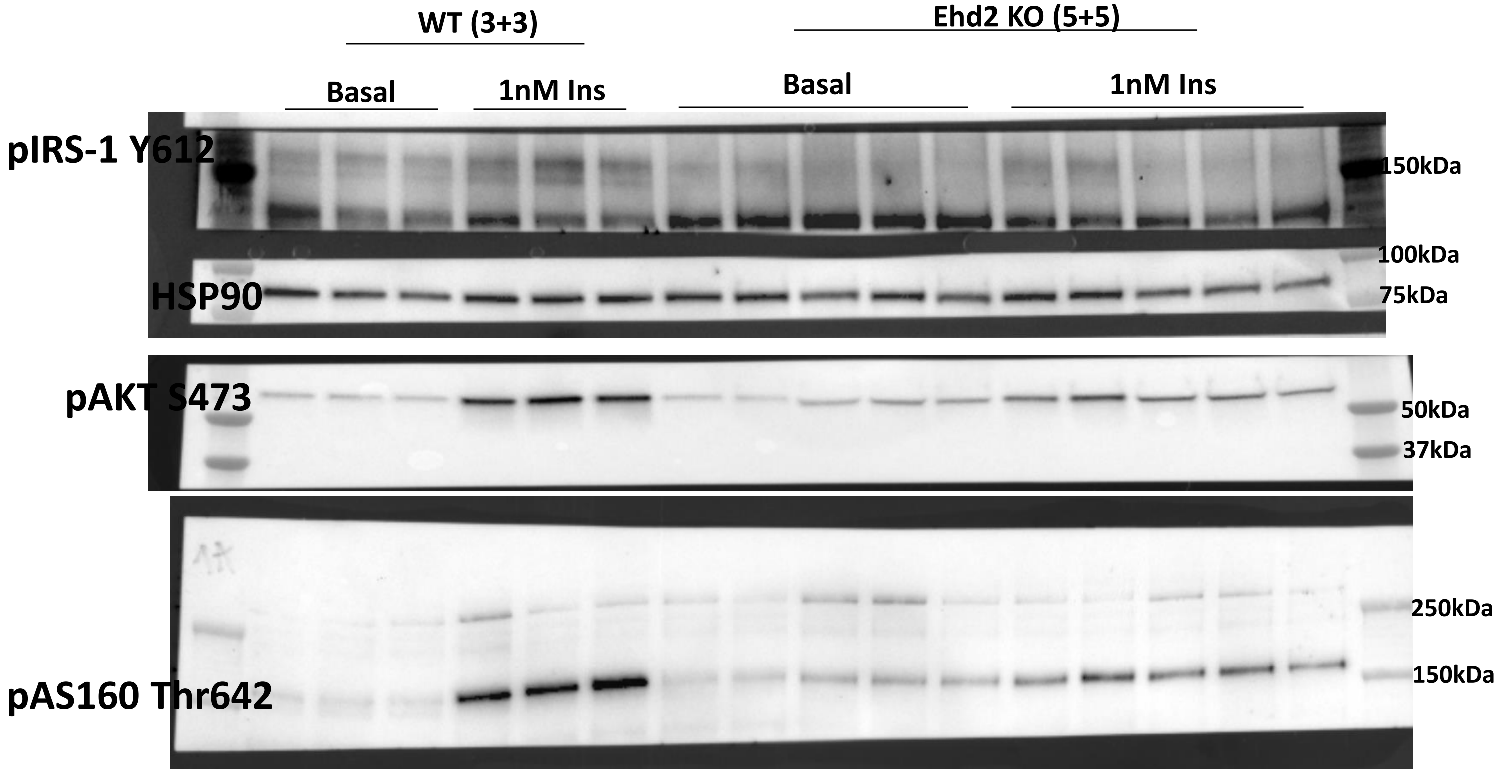

TOTAL, whole membranes for n=3 (WT) and n=5 (KO) biological replicates (ONLY Basal and 1nM)

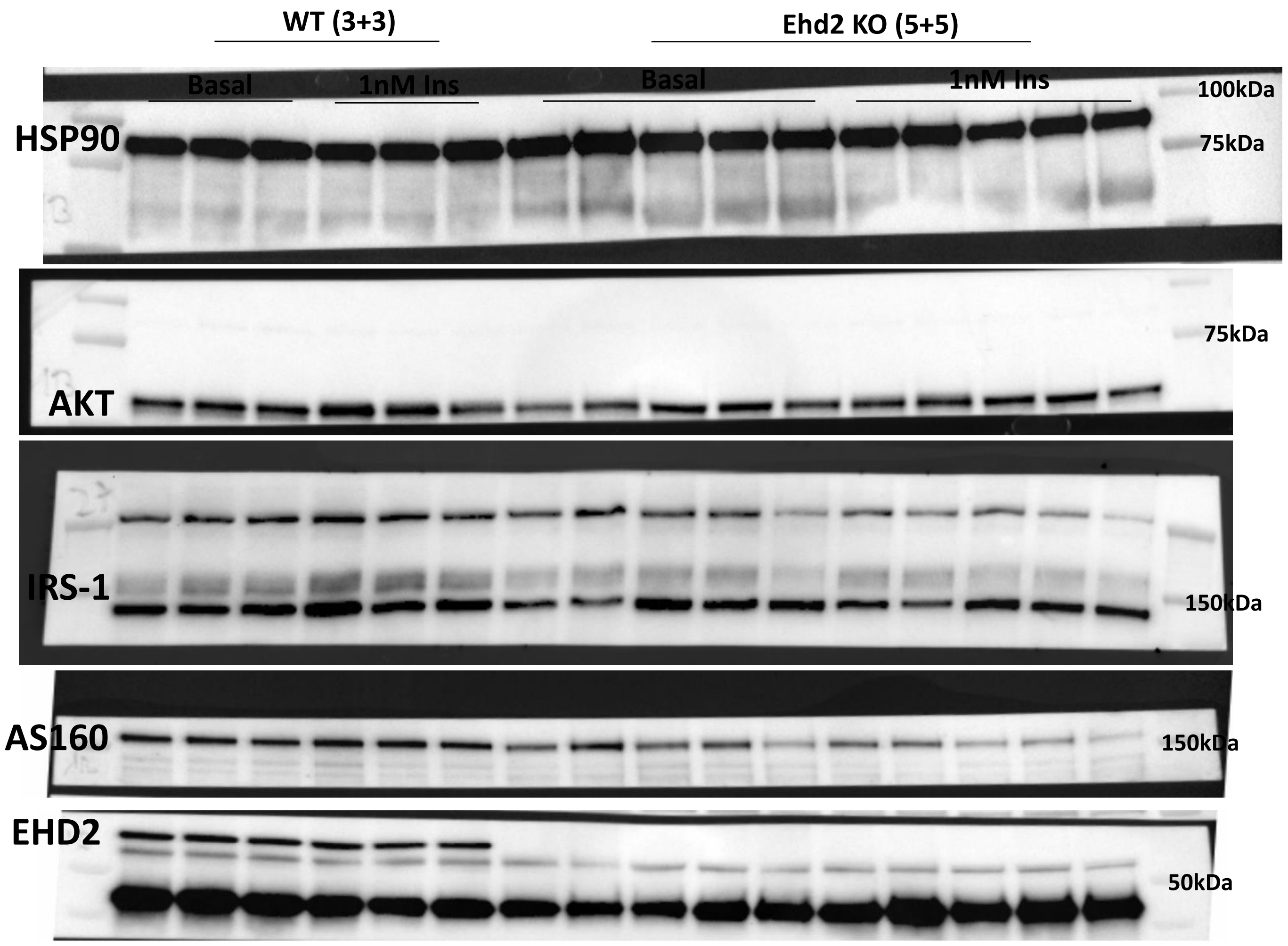

Manuscript Figure

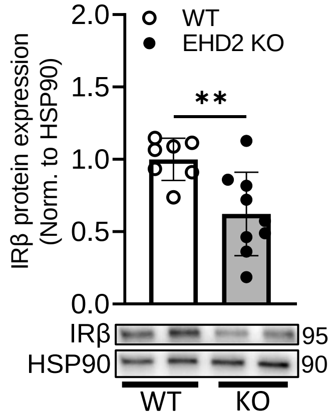

FIGURE 3A IR total levels

TOTAL IR-beta , whole membranes for n=3 (WT) and n=5 (KO) (ONLY Basal quantified)

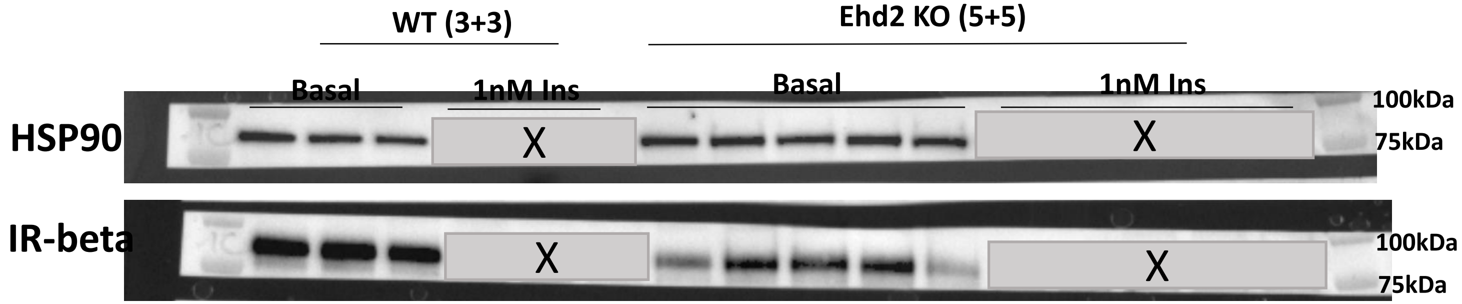

TOTAL IR-beta , whole membranes for n=4 (WT) and n=4 (KO) (ONLY Basal quantified)

MEMBRANE 1 → WT1-2 & KO1-2

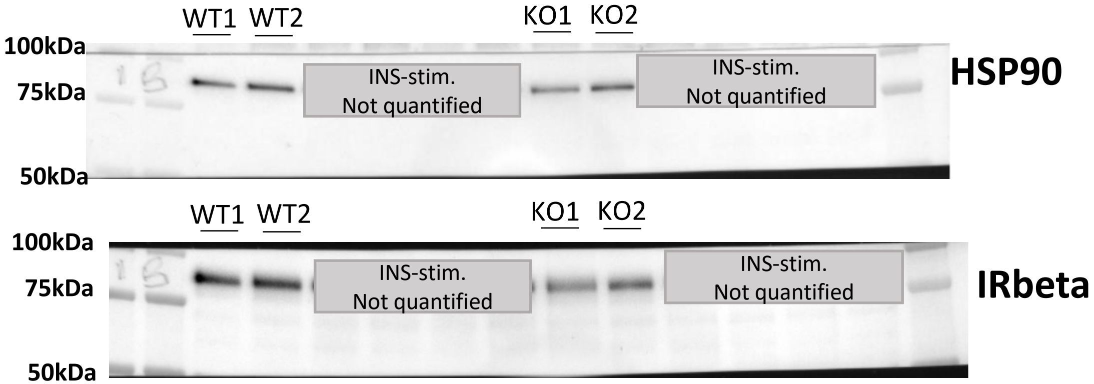

MEMBRANE 2 → WT3-4 & KO3-4

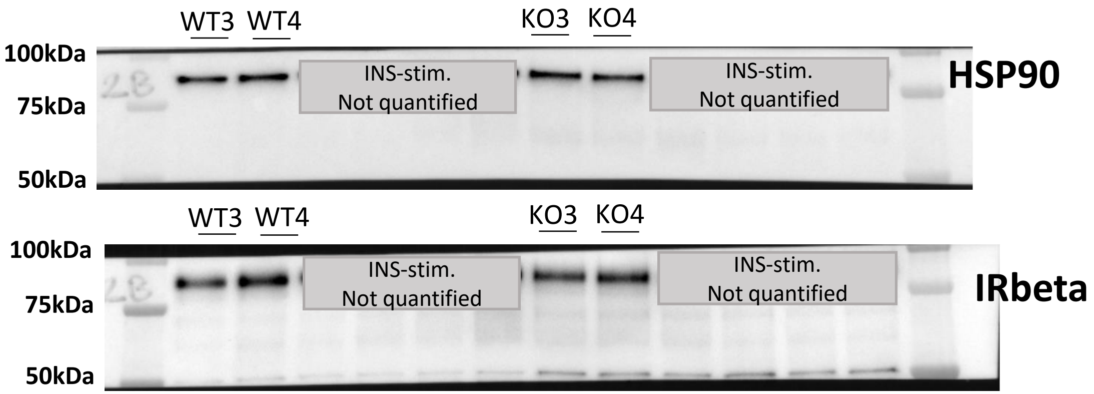

Manuscript Figure

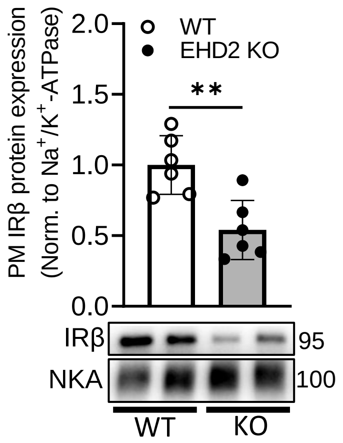

FIGURE 3B IR Plasma membrane levels

Plasma membrane IR-beta , whole membranes for n=6

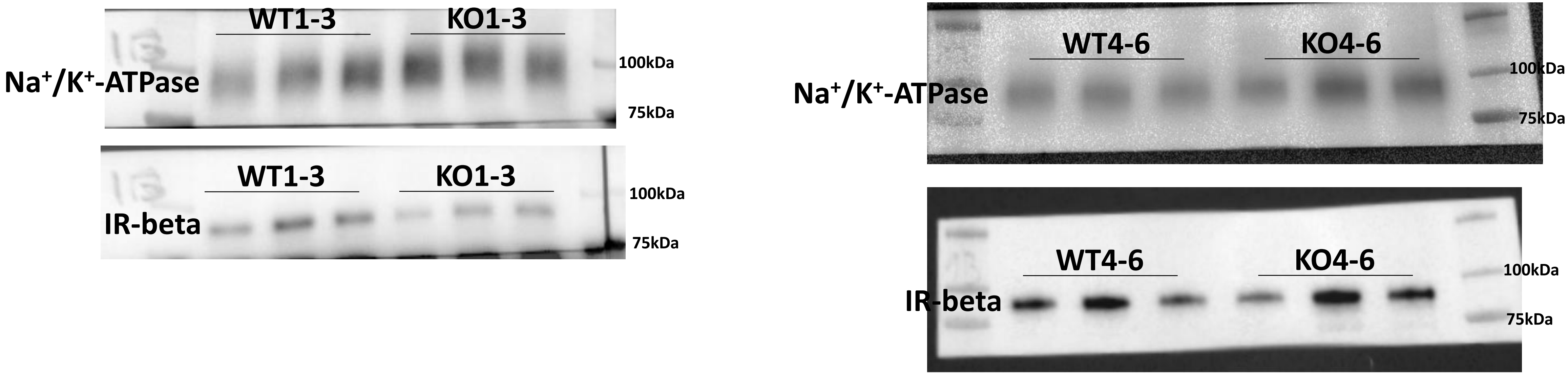

Manuscript Figure

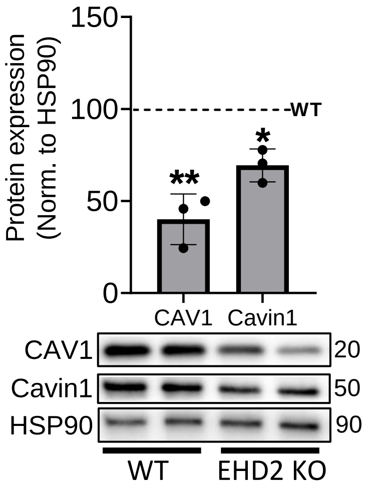

FIGURE 3D Cavin1, Caveolin-1 total levels

Total Cavin1 and Caveolin1 , whole membranes for n=3 biological replicates

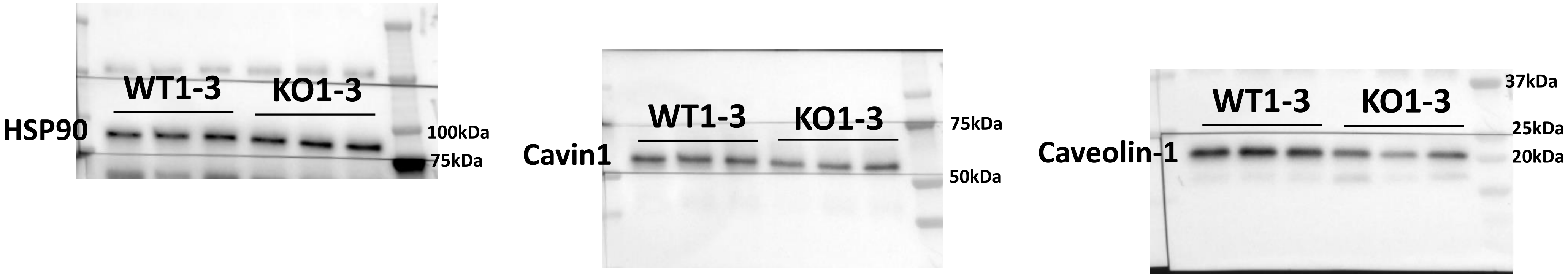

Manuscript Figure

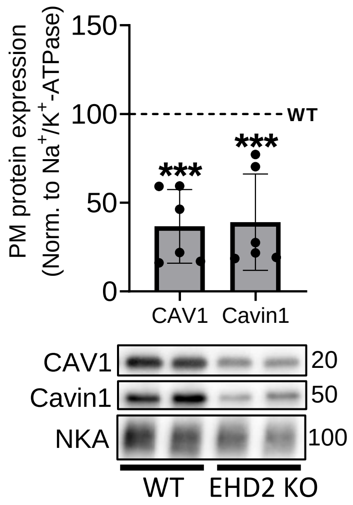

FIGURE 3E Cavin1, Caveolin-1 Plasma membrane levels

Plasma membrane Cavin1 and Caveolin1 levels; Whole membrane n=6 biological replicates

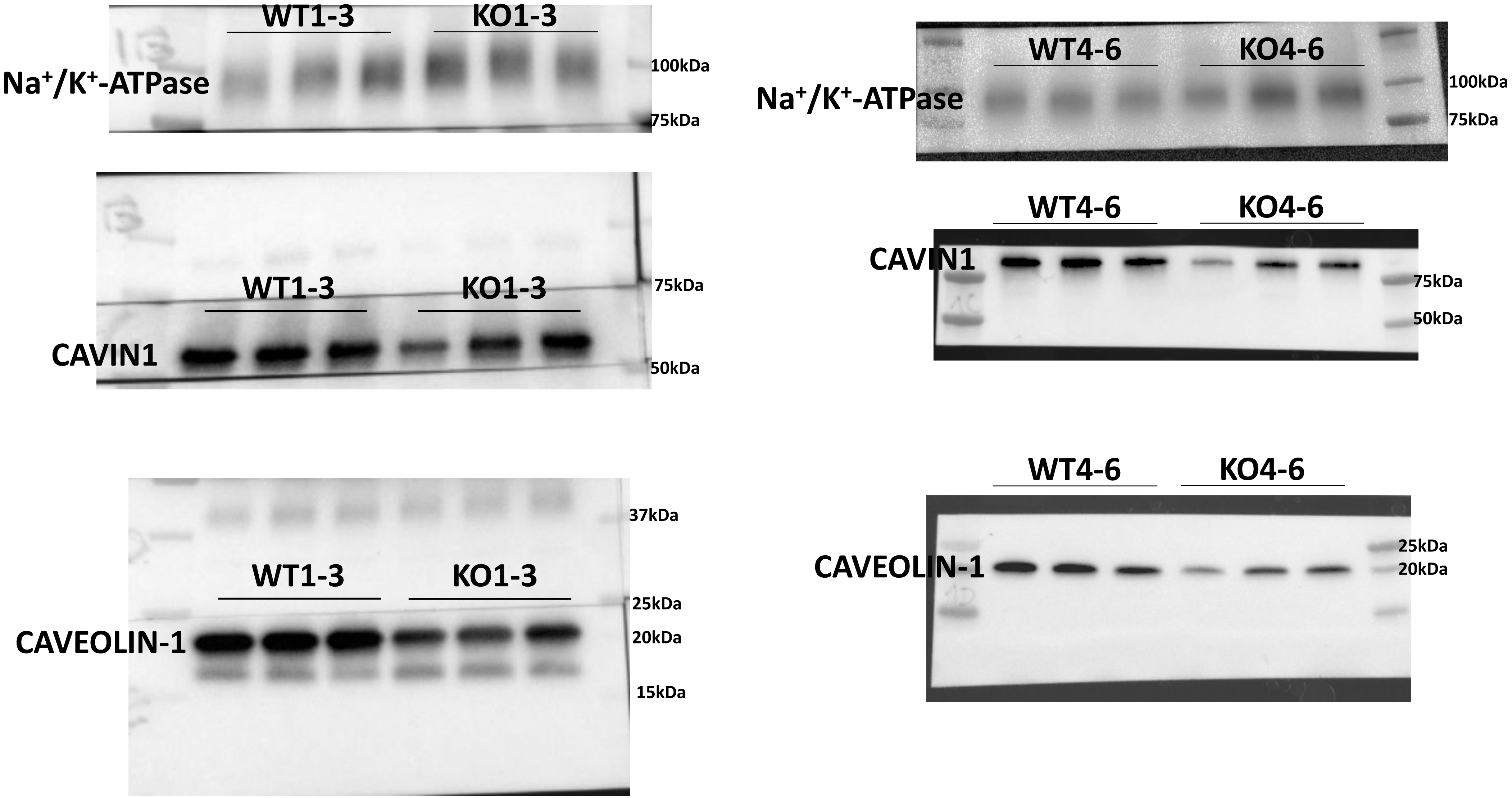

Manuscript Figure

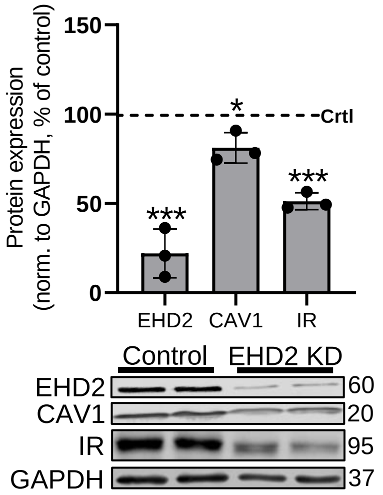

FIGURE 3F EHD2, CAV1, IR, GAPDH 3T3-L1

TOTAL CAV1, IR, GAPDH, EHD2 3T3-L1 n1 (basal quantified, total n=3 biological replicates)

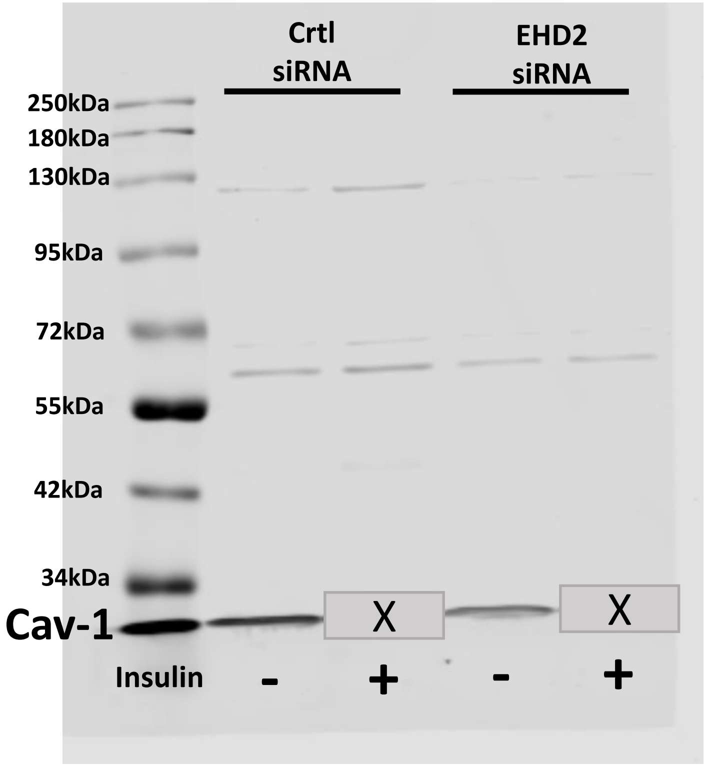

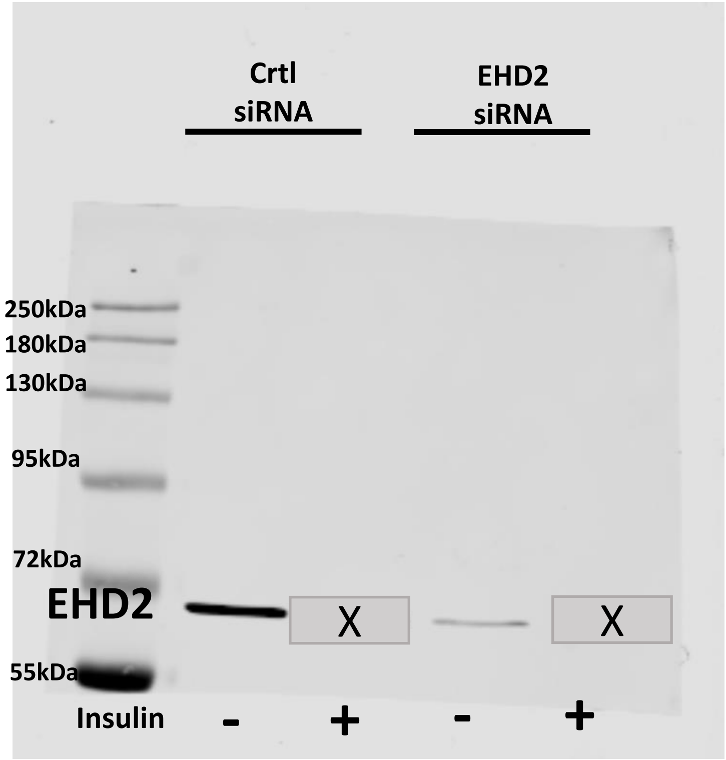

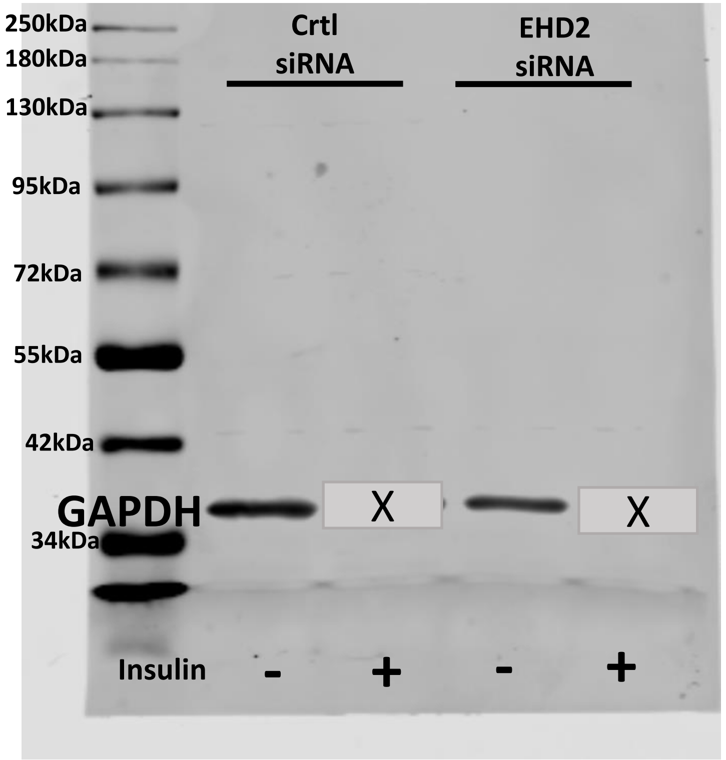

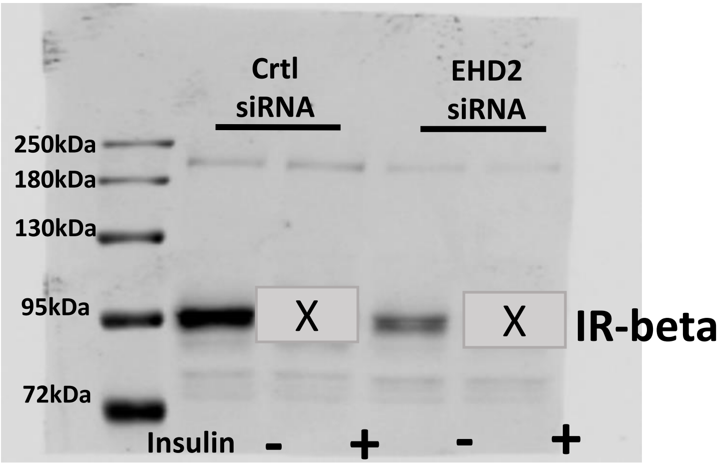

TOTAL CAV1, IR, GAPDH, EHD2 3T3-L1 n2 (total n=3 biological replicates)

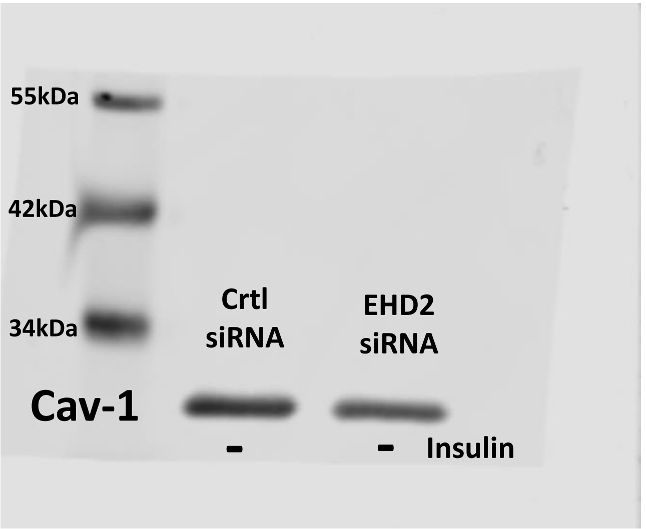

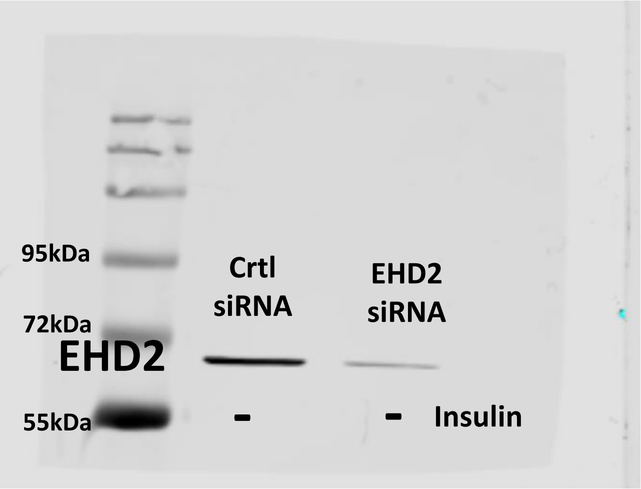

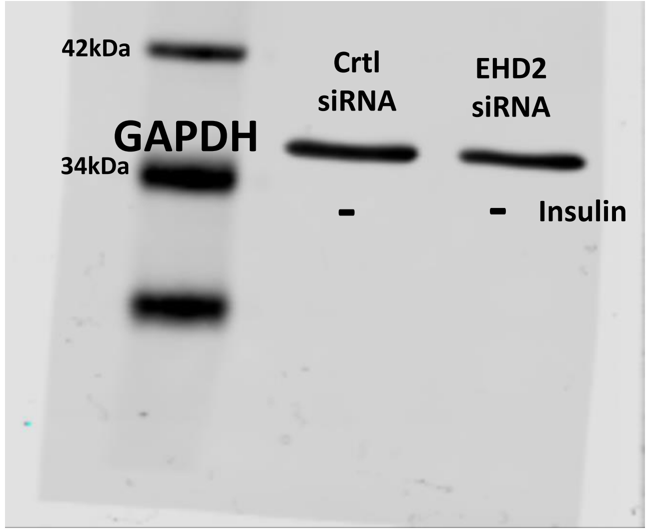

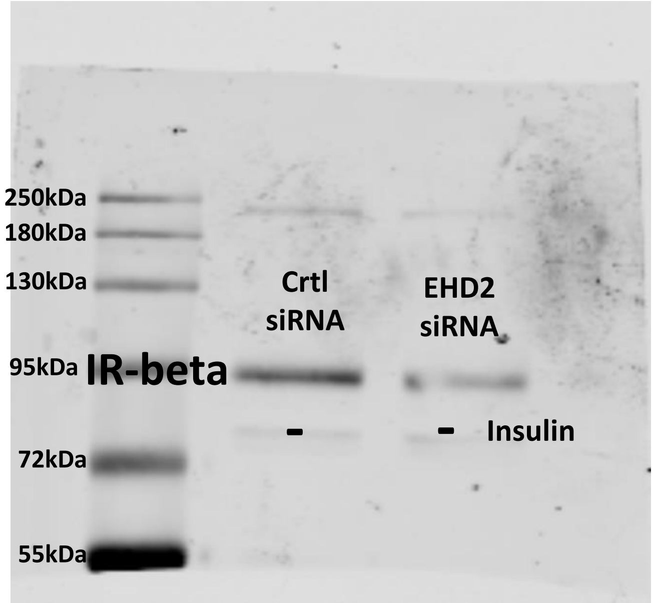

TOTAL CAV1, IR, GAPDH, EHD2 3T3-L1 n3 (basal quantified, n=3 biological replicates)

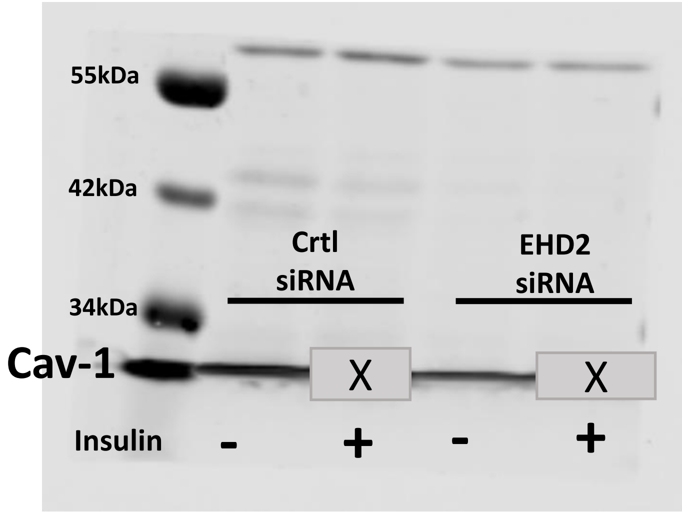

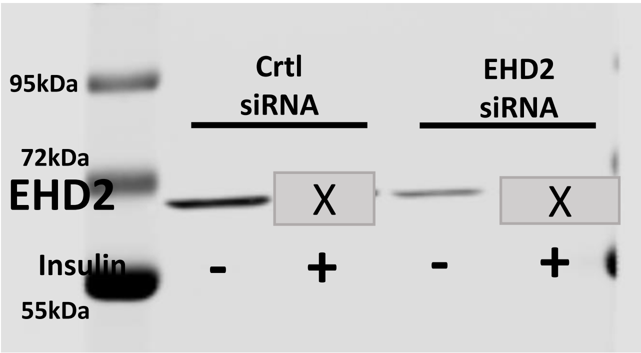

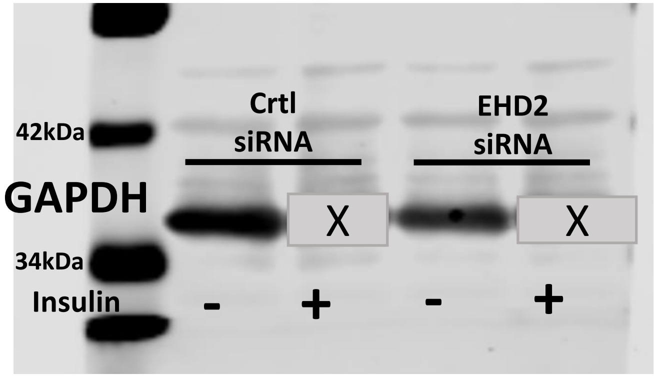

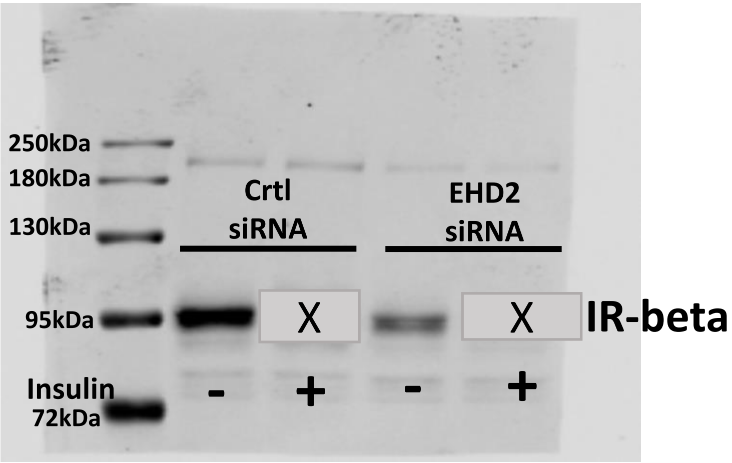

Manuscript Figure

FIGURE 4 A and C; 3T3-L1 Proteins

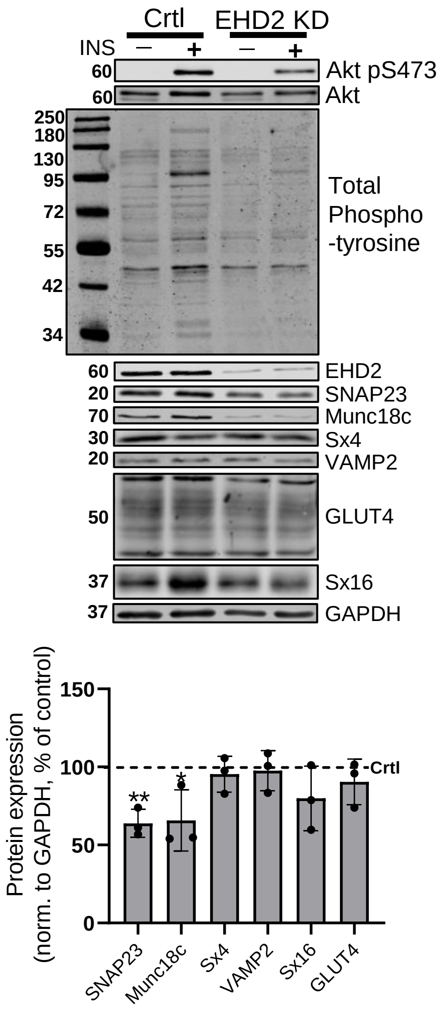

PHOSPHOTYROSINE, AKT S473 and GAPDH !n=2 biological replicates! 3T3-L1

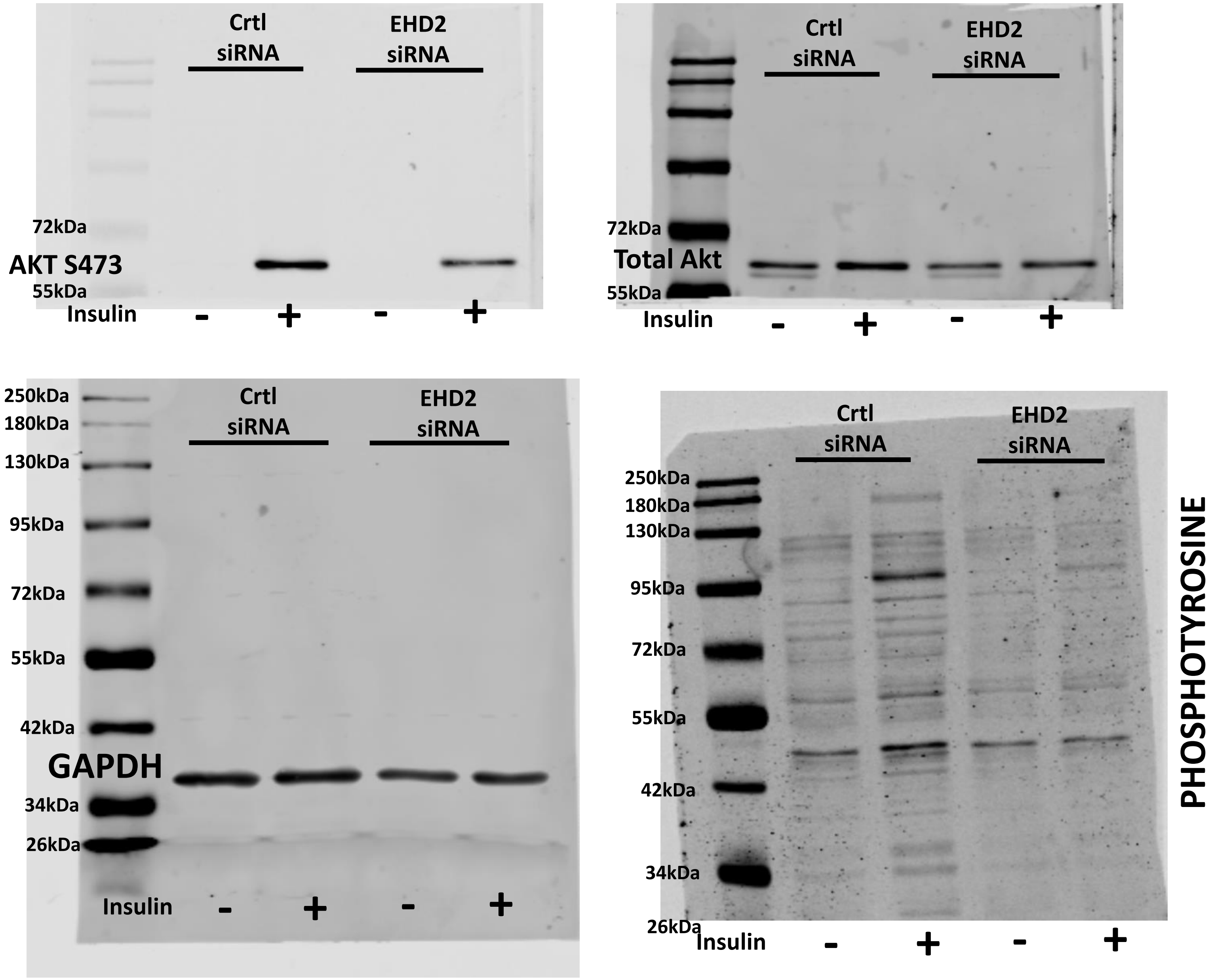

TOTAL EHD2, MUNC18c, GAPDH, GLUT4, SNAP23 3T3-L1 !n1! (basal quantified, n=3 total)

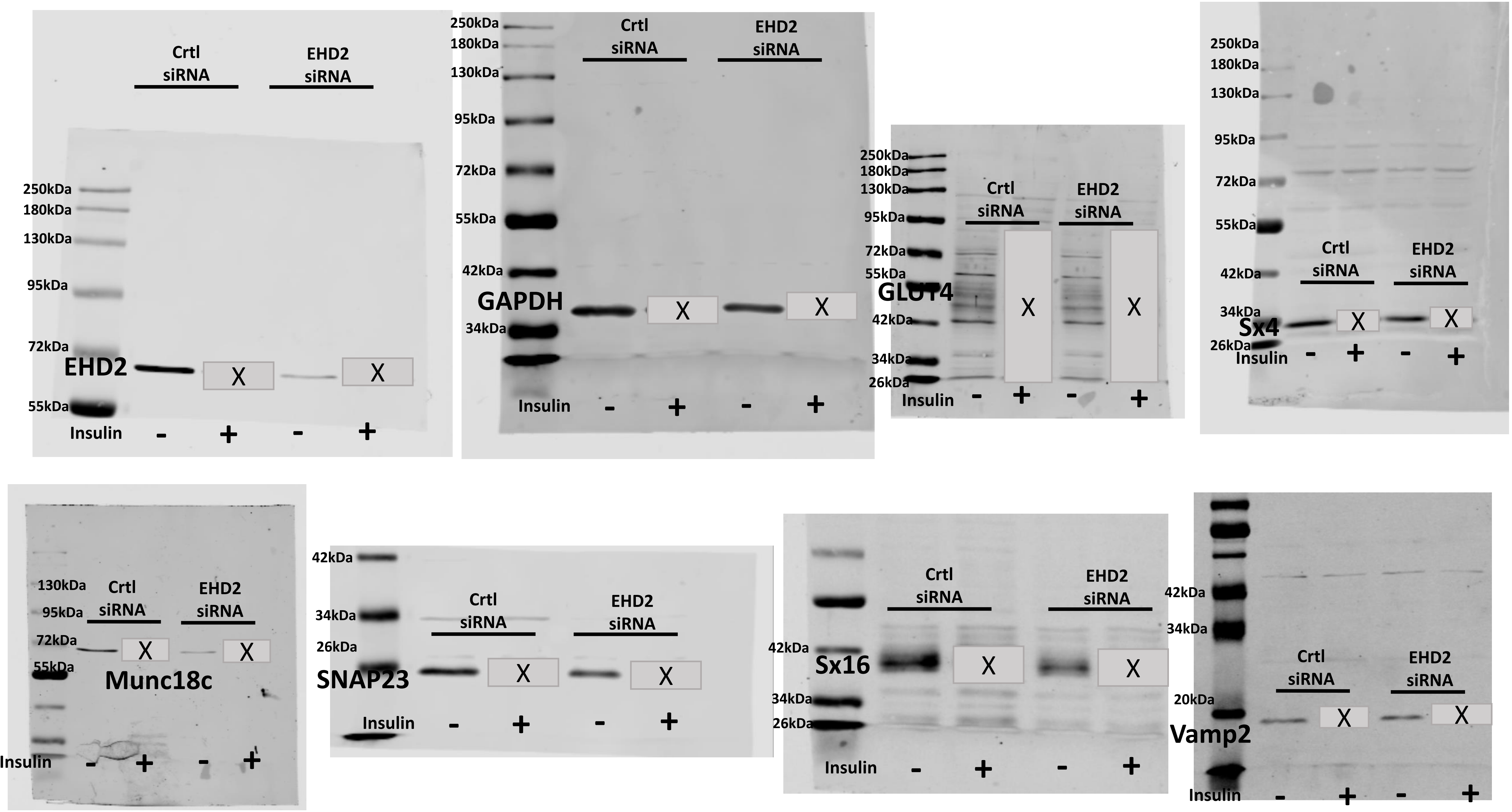

TOTAL EHD2, MUNC18c, GAPDH, GLUT4, SNAP23 3T3-L1 !n2! (basal quantified, n=3 total)

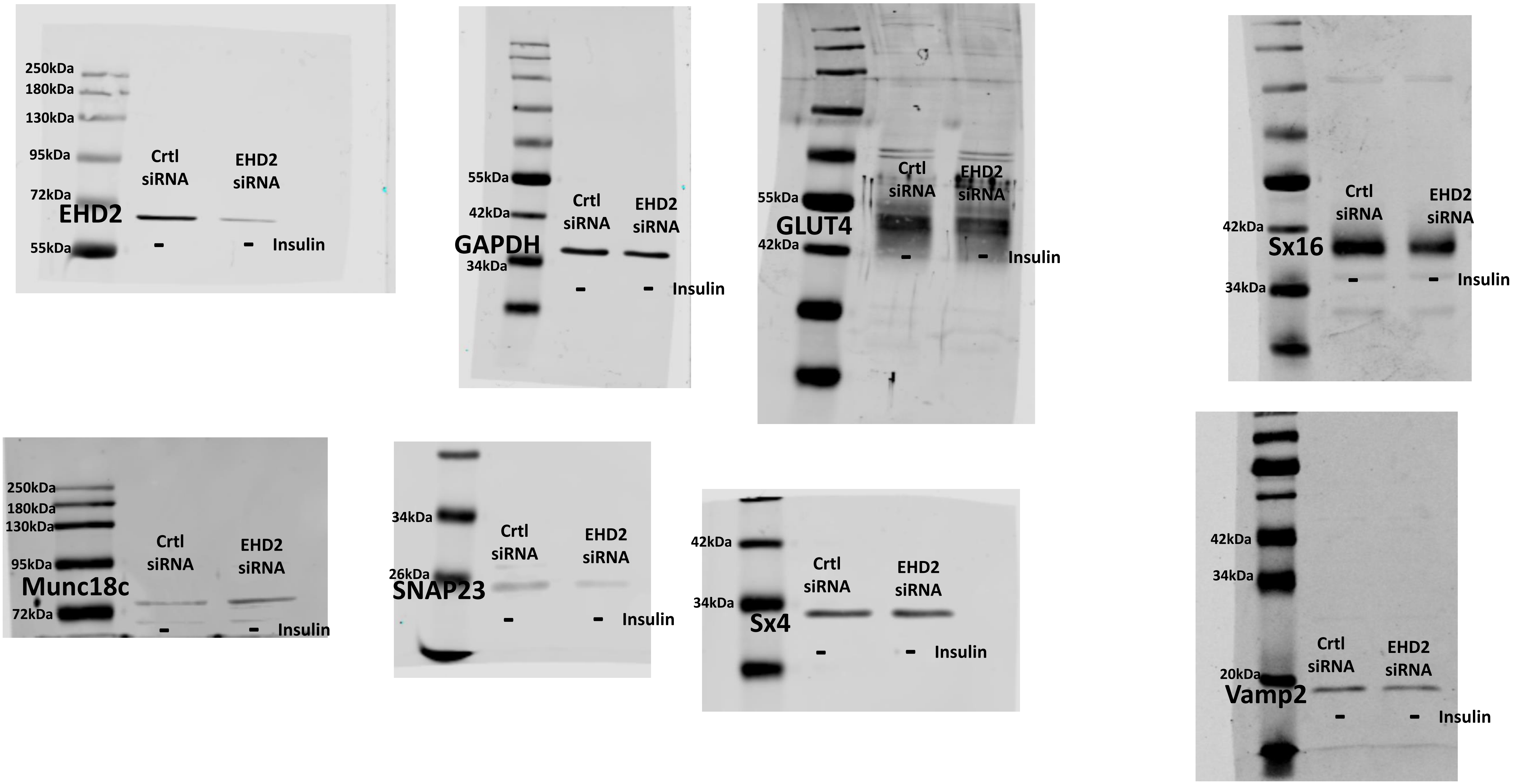

TOTAL EHD2, MUNC18c, GAPDH, GLUT4, SNAP23 3T3-L1 !n3! (basal quantified, n=3 total)

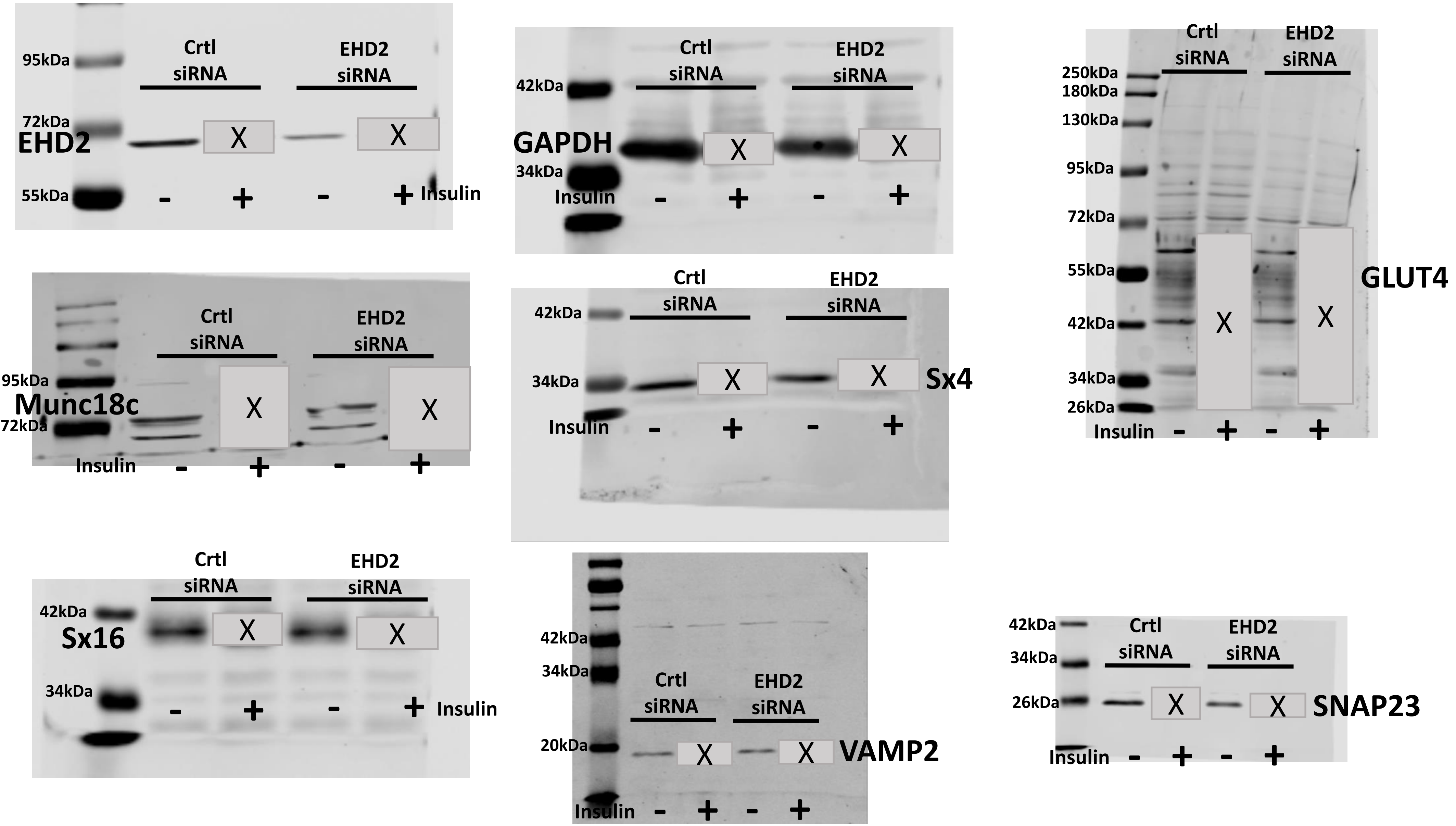

Manuscript Figure

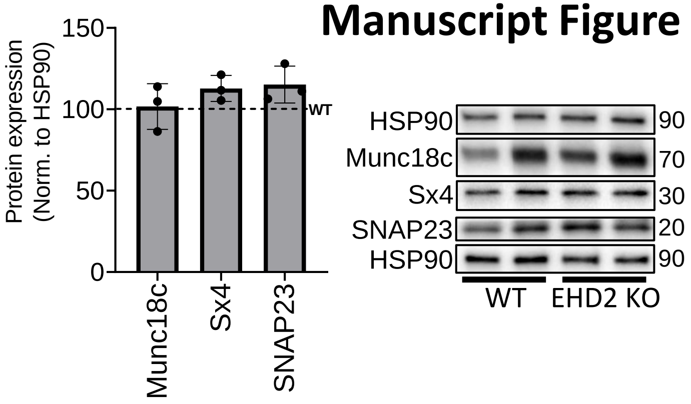

FIGURE 6 A Munc18c, Sx4, SNAP23 Total levels

Total levels MUNC18c, Sx4, SNAP23, whole membranes for n=3 biological replicates

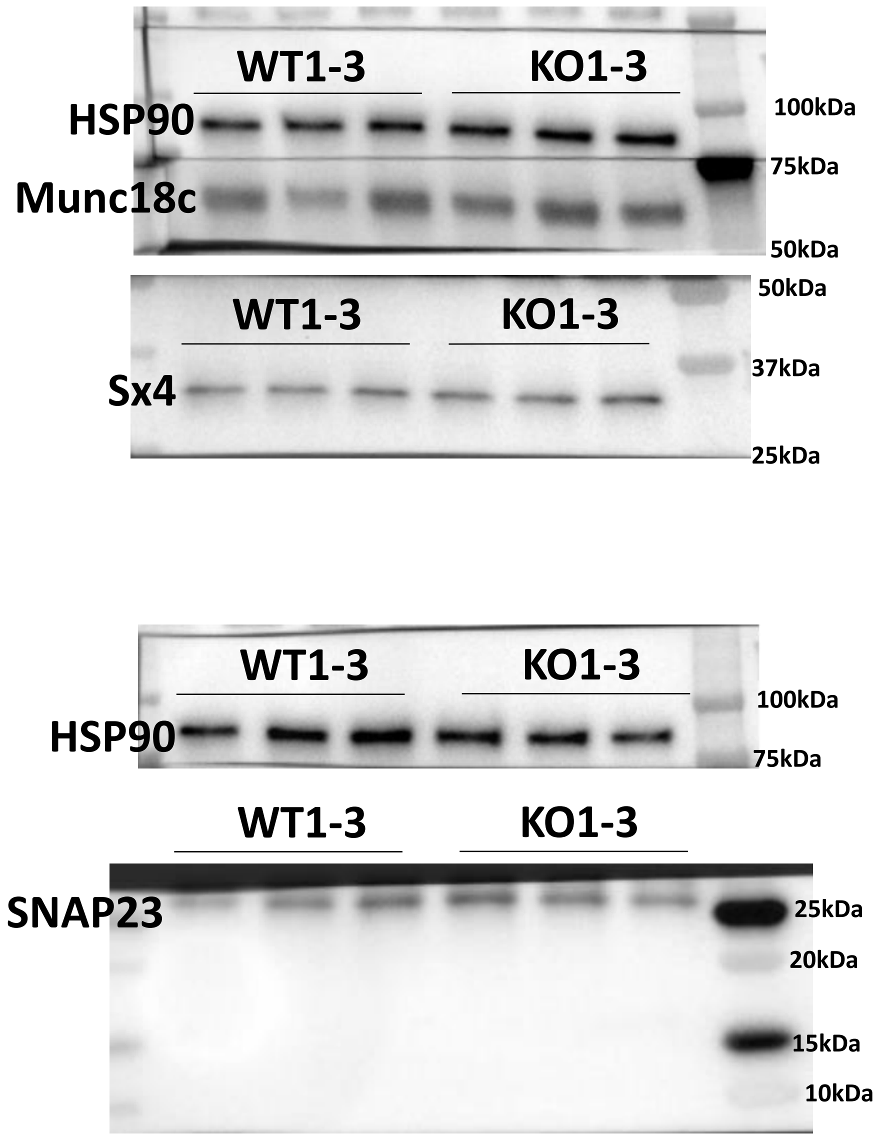

Manuscript Figure

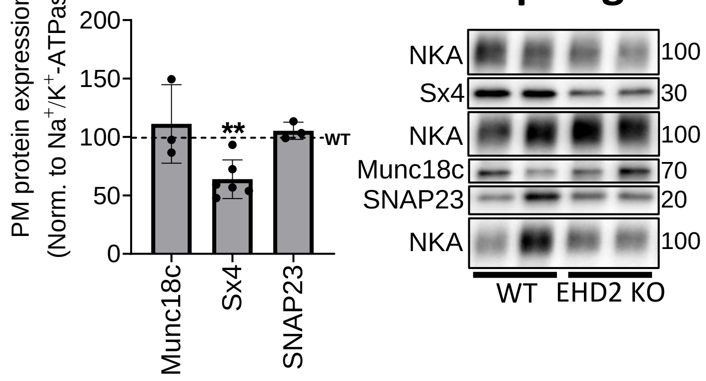

FIGURE 6 B Munc18c, Sx4, SNAP23 Plasma membrane levels

Plasma membrane levels MUNC18c, Sx4, SNAP23, whole membranes for n=3 biological replicates

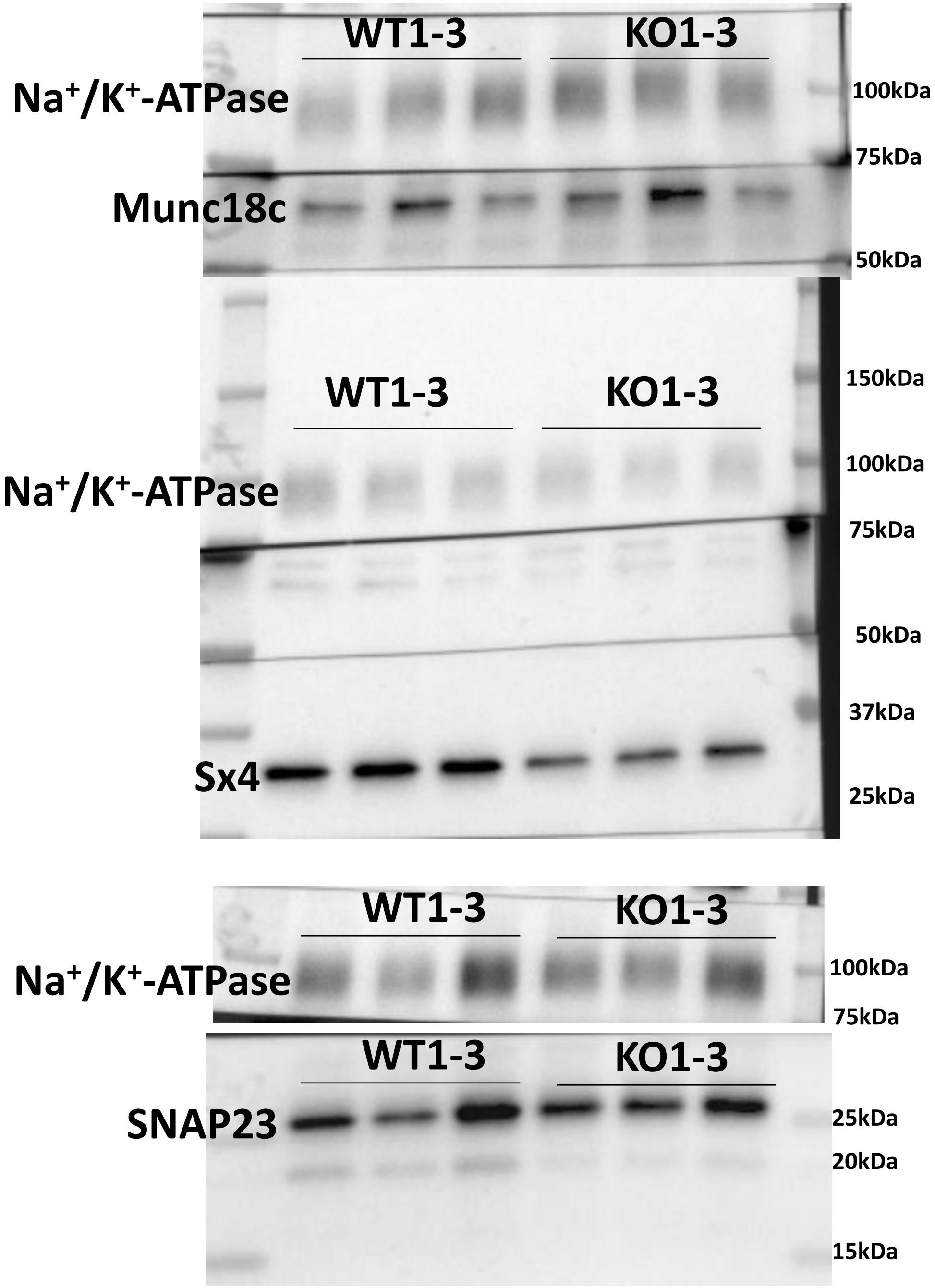

Supplement: Supplementary file 1 [file mbc-34-ar124-s001.pdf]
